# Molecular Encoding of Ischemic Stroke and its Resolution after Human Neural Stem Cell Therapy by Extracellular Vesicles

**DOI:** 10.1002/mco2.70400

**Published:** 2025-10-28

**Authors:** Chuheng Chang, Yiqing Wang, Xiaohang Liang, Renzhi Wang, Xinjie Bao

**Affiliations:** ^1^ Department of Neurosurgery Peking Union Medical College Hospital Chinese Academy of Medical Sciences & Peking Union Medical College Beijing China; ^2^ Department of Radiation Oncology National Cancer Center/National Clinical Research Center for Cancer/Cancer Hospital Chinese Academy of Medical Sciences & Peking Union Medical College Beijing China; ^3^ State Key Laboratory of Common Mechanism Research for Major Diseases Beijing China; ^4^ Department of Neurosurgery Xuanwu Hospital China International Neuroscience Institute Capital Medical University Beijing China; ^5^ Center For MRI Research Academy For Advanced Interdisciplinary Studies Peking University Beijing China; ^6^ School of Medicine Life and Health Sciences The Chinese University of Hong Kong Shenzhen Guangdong China

## Abstract

Extracellular vesicles (EVs) can cross the blood–brain barrier and enter the systemic circulation, potentially acting as peripheral biomarkers of stroke neuropathology. Here, we investigated alterations in EV RNA cargoes extracted from rat brain and plasma before and after stroke induction via middle cerebral artery occlusion and subsequent human neural stem cells (hNSCs) transplantation. EV RNA coexpression profiles were assessed, and digital source tracking was used to determine EV origin. The therapeutic effects of intra‐arterial delivery of hNSCs on ischemic rat brains were quantified, focusing on functional recovery, resolution of ischemic lesions, and the microenvironment. Stroke induced distinct EV secretion patterns, with a notable increase in EV secretion from non‐neuronal cells. hNSCs transplantation caused minimal immune rejection and transplanted cells survived in the brain for over a week. Stem cell‐derived EVs were detected in peripheral blood, indicating prolonged systemic distribution after transplantation. Gene regulatory network analyses identified specific EV miRNAs that play crucial roles in neurogenesis, wound healing, angiogenesis, and blood–brain barrier integrity. An integrated analysis of EV RNAs in brain and plasma samples revealed that stroke increased correlations in RNA expression between brain and plasma and that hNSCs transplantation reversed the effect. Brain‐ and plasma‐derived EVs carry similar molecular information after stroke, suggesting that plasma‐derived EV RNAs reflect stroke pathophysiology. Intra‐arterial transplantation of hNSCs improved outcomes after stroke in rats, by promoting endogenous neurogenesis and maintaining blood–brain barrier integrity. The identified EV miRNAs provided a new mechanism by which hNSCs transplantation regulates neural regeneration through the miR‐204‐5p/EFNB3 axis.

## Graphical abstract

EVs can cross the blood–brain barrier and enter systemic circulation. By analyzing EV RNA cargoes before and after stroke, and following hNSC transplantation, our study shows that EV RNAs accurately reflect stroke pathology and provide insights into how hNSCs promote stroke recovery. These findings suggest that EV RNA profiles could serve as diagnostic and predictive biomarkers for stroke and recovery.

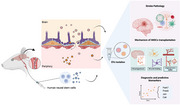

## 1 Introduction

Extracellular vesicles (EVs) are bilayered lipid nanoparticles arising from many cell types that play pivotal roles in waste disposal, signal transduction, and tumorigenesis [1]. Neurons and other diverse non‐neuronal cells including microglia, astrocytes, and endothelial cells secrete EVs in the brain [2]. EVs originating from the central nervous system (CNS) can cross the blood–brain barrier and enter the peripheral circulation, which, in pathological conditions, carry brain‐specific biomarkers of diseases including neurodegenerative disorders (Alzheimer's disease, Parkinson's disease) and stroke [3, 4, 5]. The presence of these biomarkers reflects neuroinflammatory responses and cytotoxic alterations within the CNS, offering a systemic window into the pathological processes occurring within the brain.

Ischemic stroke, which primarily arises from occlusion of cerebral blood vessels by thrombus, is the most common type of stroke and it frequently leads to chronic neurological dysfunction associated with poor clinical outcomes [6]. The administration of recombinant tissue plasminogen activator (rt‐PA) limits infarct volume and promotes neurological recovery, but its application is limited by a restricted therapeutic window and contraindications [7]. Neural stem cells (NSCs) transplantation might be a feasible means to promote neural repair and regeneration [8, 9]. The PISCES trial series has provided supporting evidence for the safety and potential therapeutic efficacy of human (h)NSCs‐based treatment [10, 11]. Our previous work in primate stroke models showed that transplantation of human NSCs (hNSCs) improves motor function and reduces infarct volume in the animal model [12]. Mechanistic investigations revealed that hNSCs promote functional recovery through restoring mitochondrial function, enhancing neurogenesis, and modulating immune responses.

To support preclinical and clinical trials, biomarkers to monitor the efficacy of cell‐based therapy and guide practice are required. EVs secreted by NSCs contain proneural miR‐9 and anti‐inflammatory miR‐7, which benefit stroke recovery [13, 14]. Notably, miR‐124, a highly abundant brain miRNA, demonstrated differential responses in distinct serum‐derived fractions following acute ischemic stroke. Specifically, miR‐124 isolated from serum EVs exhibited greater sensitivity to stroke onset than miR‐124 measured in whole serum [15]. Both serum EV‐derived and whole‐serum miR‐124 levels declined after the acute event; however, only the EV‐associated miR‐124 levels were restored following rt‐PA treatment, whereas whole‐serum miR‐124 levels remained unchanged. These observations highlight the potential of EV‐derived RNAs to more accurately reflect dynamic pathophysiological changes and to serve as sensitive biomarkers for evaluating therapeutic responses, including hNSCs transplantation.

Here, we examined alterations in EV RNA cargoes isolated from rat brain and plasma before and after stroke induced by middle cerebral artery occlusion (MCAO). We analyzed the coexpression profiles of EV RNAs and used a digital source tracking technique to establish the origins of EVs in the brain and plasma. Furthermore, we assessed the therapeutic impact of intra‐arterial human hNSCs transplantation and associated alterations in EV RNAs following transplantation. We showed that EV miRNAs play a pivotal role in stroke recovery by modulating neurogenesis, angiogenesis, and endothelial barrier function. Among the significantly enriched miRNAs, we identified miR‐204‐5p as a potential regulator of neurogenesis, acting through the modulation of EFNB3.

## 2 Results

### 2.1 Brain‐Derived EV RNA Expression Profiles after Stroke

**FIGURE 1:**
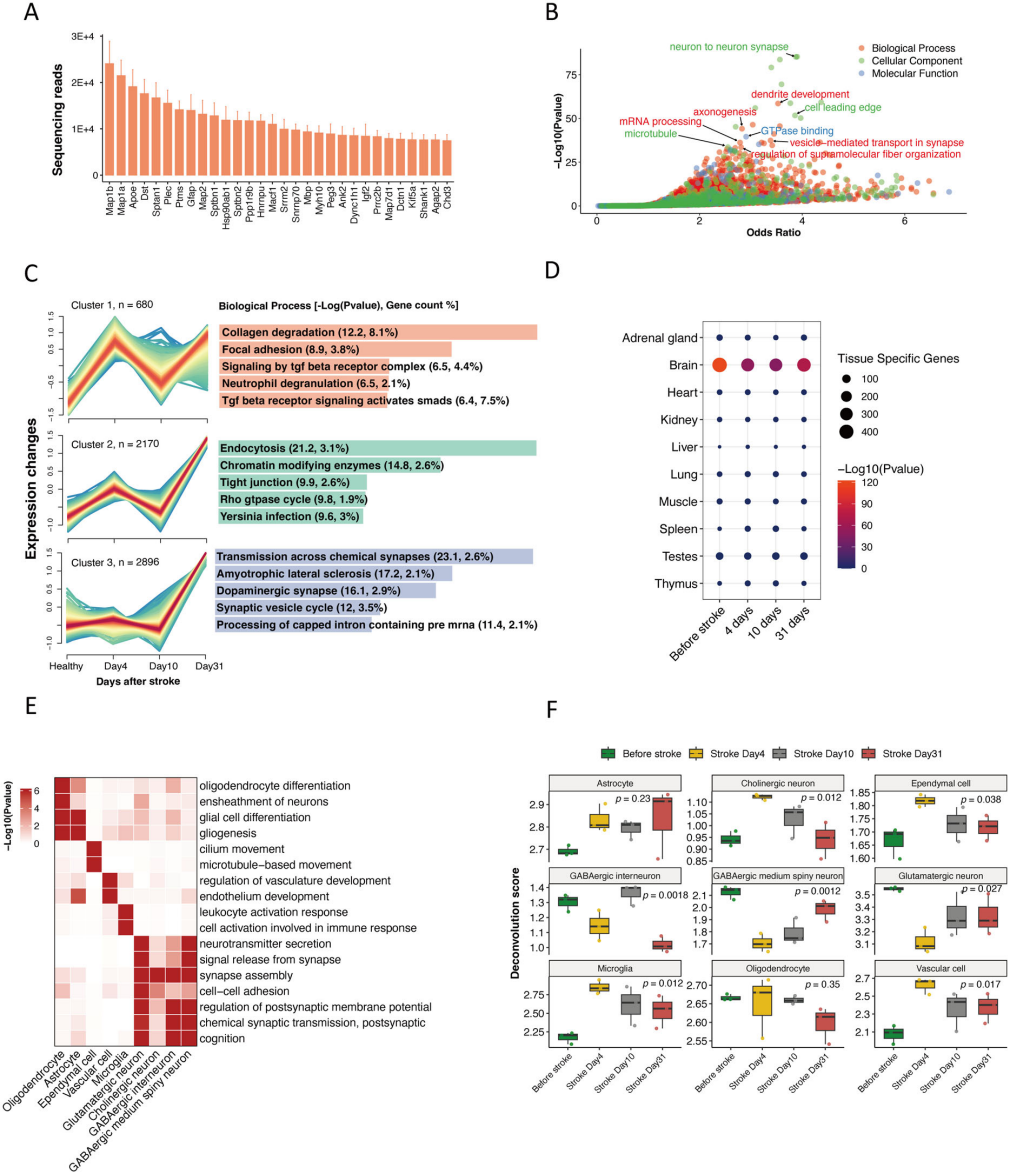
Source tracking of brain‐derived EVs. (A) The top 30 most abundant rat brain‐derived EV mRNA cargoes. (B) Gene ontology enrichment dot plot of the top 3000 expressed mRNAs in brain‐derived EVs. (C) Coexpression analysis of mRNAs in brain‐derived EVs. The left panel depicts cluster trajectories, and the right panel illustrates the corresponding pathway annotations for each cluster. Each trajectory line represents an individual gene, and the color gradient from red to blue indicates the degree of conformity of genes to the central trajectory. (D) Tissue enrichment analysis of brain‐derived EVs. The size of each circle represents the number of tissue‐specific genes present in the corresponding tissue, and the color gradient from red to blue reflects the statistical significance. (E) Functional enrichment of the cell type‐specific genes from the signature matrix. (F) Source tracking analysis based on deconvolution of brain‐derived EVs. *n* = 3 animals per group. Differences observed within distinct cell types across various time points were evaluated by analysis of variance.

Stroke was established in rats by transient occlusion (90 min) of the middle cerebral artery. Rats were euthanized for extraction of EV RNA from ipsilateral hemisphere brain tissues 4, 10, and 31 days after stroke (Figure S1A–C). Microtubule‐associated protein‐1b and 1a (Map1b/a) were the most abundantly expressed transcripts (Figure 1A), which are highly concentrated in dendritic spines and can be secreted into the extracellular space via EVs during neuronal depolarization [16]. Gene ontology analysis revealed that brain EV mRNAs were involved in dendrite development and axonogenesis and vesicle‐mediated transport in synapses (Figure 1B).

Coexpression analysis revealed three distinct mRNA expression temporal trajectories (Figures 1C and S1D and Table S1). Genes within cluster 1 were highly expressed 4 days after stroke, followed by attenuation at 10 days and a subsequent surge at 31 days, containing representation of collagen degradation, focal adhesion, neutrophil degranulation, and TGF‐β signaling pathways. Expression levels in cluster 2 were similar to cluster 1; however, expression at 31 days was notably higher than at day 4. Pathways associated with this cluster were related to endocytosis, chromatin modification, tight junctions, and the Rho GTPase cycle. Genes within cluster 3 were elevated exclusively at 31 days poststroke and were involved in chemical synapse transmission, dopaminergic synapses, synaptic vesicle cycle, and capped intron‐containing pre‐mRNA processing pathways.

To evaluate the tissue origin of EVs, we constructed a signature matrix incorporating tissue‐specific genes (TSGs) unique to 10 organs: brain, heart, kidney, adrenal gland, liver, lung, muscle, spleen, testes, and thymus. As expected, enrichment scores were highest for brain TSGs (Figure 1D). Notably, brain‐specific TSG scores in rats before stroke surgery were higher than those after stroke, suggesting infiltration of EVs originating from nonbrain tissues into the CNS after stroke.

To dissect the distinct brain cell types responsible for the production of brain‐derived EVs, we created a customized cell matrix incorporating different brain cell types including oligodendrocyte, astrocyte, ependymal cell, vascular cell, microglia, GABAergic medium spiny neuron, GABAergic interneuron, cholinergic neuron, and glutamatergic neuron (Figures 1E and S1E,F and Table S2). Under physiological conditions, glutamatergic excitatory neurons were the primary sources of EVs. However, 4 days after stroke, neuronal EV secretion decreased, while non‐neuronal cells—particularly microglia, vascular cells, and ependymal cells—increased their EV secretion (Figure 1F). Interestingly, cholinergic neurons were the only neuronal subtype to exhibit upregulated EV secretion poststroke. Notably, stroke‐induced changes in EV secretion gradually returned to baseline levels in subsequent weeks.

### 2.2 Expression Profiles of Plasma‐Derived EV RNAs after Stroke

**FIGURE 2:**
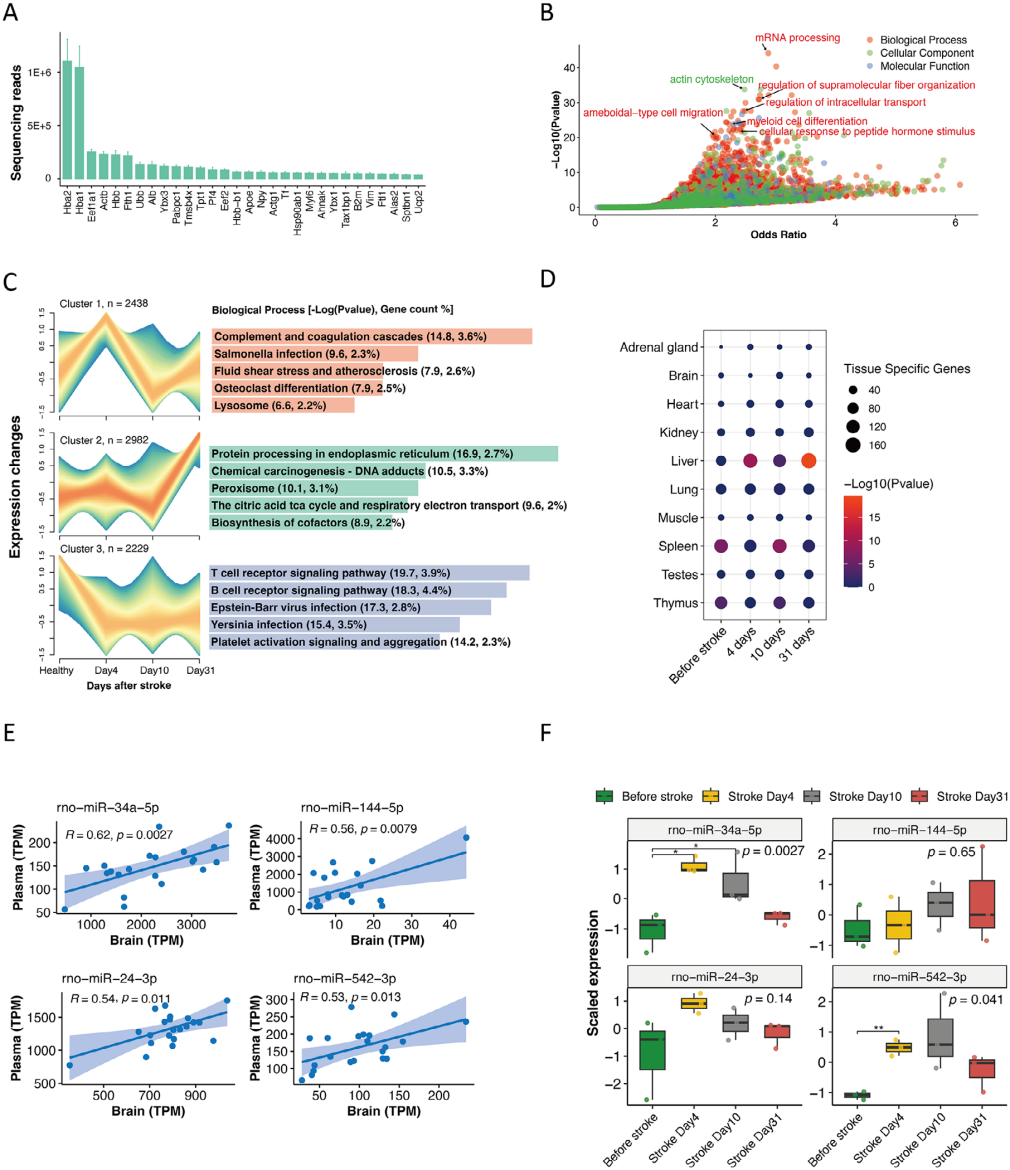
Source tracking of plasma‐derived EVs. (A) The top 30 most abundant rats plasma‐derived EV mRNA cargoes. (B) Gene ontology enrichment dot plot of the top 3000 expressed mRNAs in the plasma‐derived EVs. (C) Coexpression analysis of mRNAs in plasma‐derived EVs. The left panel depicts cluster trajectories, and the right panel illustrates the corresponding pathway annotations for each cluster. Each trajectory line represents an individual gene, and the color gradient from red to blue indicates the degree of conformity of genes to the central trajectory. (D) Tissue enrichment analysis of plasma‐derived EVs. The size of each circle represents the number of tissue‐specific genes present in the corresponding tissue, and the color gradient from red to blue reflects the statistical significance. (E) Correlation plots of the four miRNAs with the highest Pearson correlations between the brain and plasma. (F) Boxplot showing the expression levels of the four miRNAs in healthy controls versus stroke groups at 4, 10, and 31 days poststroke. For multigroup comparisons, we applied analysis of variance, while Student's *t*‐test was used for pairwise comparisons between stroke time points and the healthy control group. *p < 0.05, **p < 0.01.

Next, analysis of RNA enrichment in plasma‐derived EVs revealed that hemoglobin subunit α 2 and 1 (Hba2/1) were the most abundant mRNAs in plasma‐derived EVs (Figure 2A). Enrichment analysis revealed that plasma‐derived EV mRNAs were related to the cellular component of the actin cytoskeleton and were involved in the biological processes of fiber organization, intracellular transport, myeloid cell differentiation, and ameboid‐type cell migration (Figure 2B). Three distinct temporal trajectories were identified in the coexpression analysis (Figures 2C and S1G and Table S1). Cluster 1 contained genes peaking in expression on day 4 after stroke and participating in pathways related to complement and coagulation cascades, atherosclerosis, and osteoclast differentiation. Cluster 2 genes were upregulated at 31 days and were involved in pathways associated with endoplasmic reticulum protein processing, citric acid TCA cycle, respiratory electron transportation, and cofactor biosynthesis. Cluster 3 contained genes that were downregulated following stroke and maintained low expression levels for over a month. These genes were linked to lymphocyte receptor signaling and platelet activation. Tissue enrichment analysis revealed that the liver and spleen were the primary sources of plasma EVs (Figure 2D).

To examine whether plasma EV miRNAs could reflect the expression changes of their brain counterparts, we compared their expression levels between brain and plasma. The top four most correlated miRNAs between brain and plasma were miR‐34a‐5p, miR‐144‐5p, miR‐24‐3p, and miR‐542‐3p (Figure 2E). We found that miR‐34a‐5p and miR‐542‐3p distinguished stroke groups from healthy groups 4 days after stroke, gradually returning to baseline levels by day 31. Notably, miR‐34a‐5p remained stably elevated at 10 days poststroke, suggesting its potential as a biomarker within this early poststroke period (Figure 2F). This finding suggests these two peripheral miRNAs could serve as indicators of the CNS and might also be useful biomarkers of ischemic stroke. Additionally, we also compared mRNA expression levels between plasma‐derived EVs and brain‐derived EVs (Figure S1H and Table S3). While some of these miRNAs and mRNAs showed good correlation between plasma and brain‐derived EVs, miRNAs were significantly more abundant. Previous studies have demonstrated that cells secrete EVs of different sizes, such as microvesicles and exosomes. For microvesicles larger than 200 nm, mRNA is more abundant, while for exosomes with diameters between 100 and 200 nm, miRNAs are more abundant and stable [17]. In this study, our extracted EVs were about 100–200 nm in size (Figure S1C). Thus, EV‐miRNAs, rather than EV‐mRNAs, enriched by our method may be more stably expressed.

We compare our results with previous studies that examined the small RNA sequencing of plasma EVs from patients with stroke [18]. Among these, rno‐miR‐532‐3p, rno‐miR‐15b‐3p, rno‐miR‐423‐5p, and rno‐miR‐155‐5p, which correspond to their human counterparts (hsa‐miR‐532‐3p, hsa‐miR‐15b‐3p, hsa‐miR‐423‐5p, and hsa‐miR‐155‐5p), were found to be downregulated in both datasets. Additionally, rno‐miR‐181b‐1‐3p and rno‐miR‐29a‐5p, corresponding to hsa‐miR‐181b‐1‐3p and hsa‐miR‐29a‐5p, were upregulated in both datasets. These findings highlight an overlap in EV‐miRNA expression patterns between the two studies, suggesting conserved molecular mechanisms in stroke pathogenesis and potential cross‐species biomarkers for stroke diagnosis.

### 2.3 hNSCs Transplantation Promotes Stroke Recovery in Rats

**FIGURE 3:**
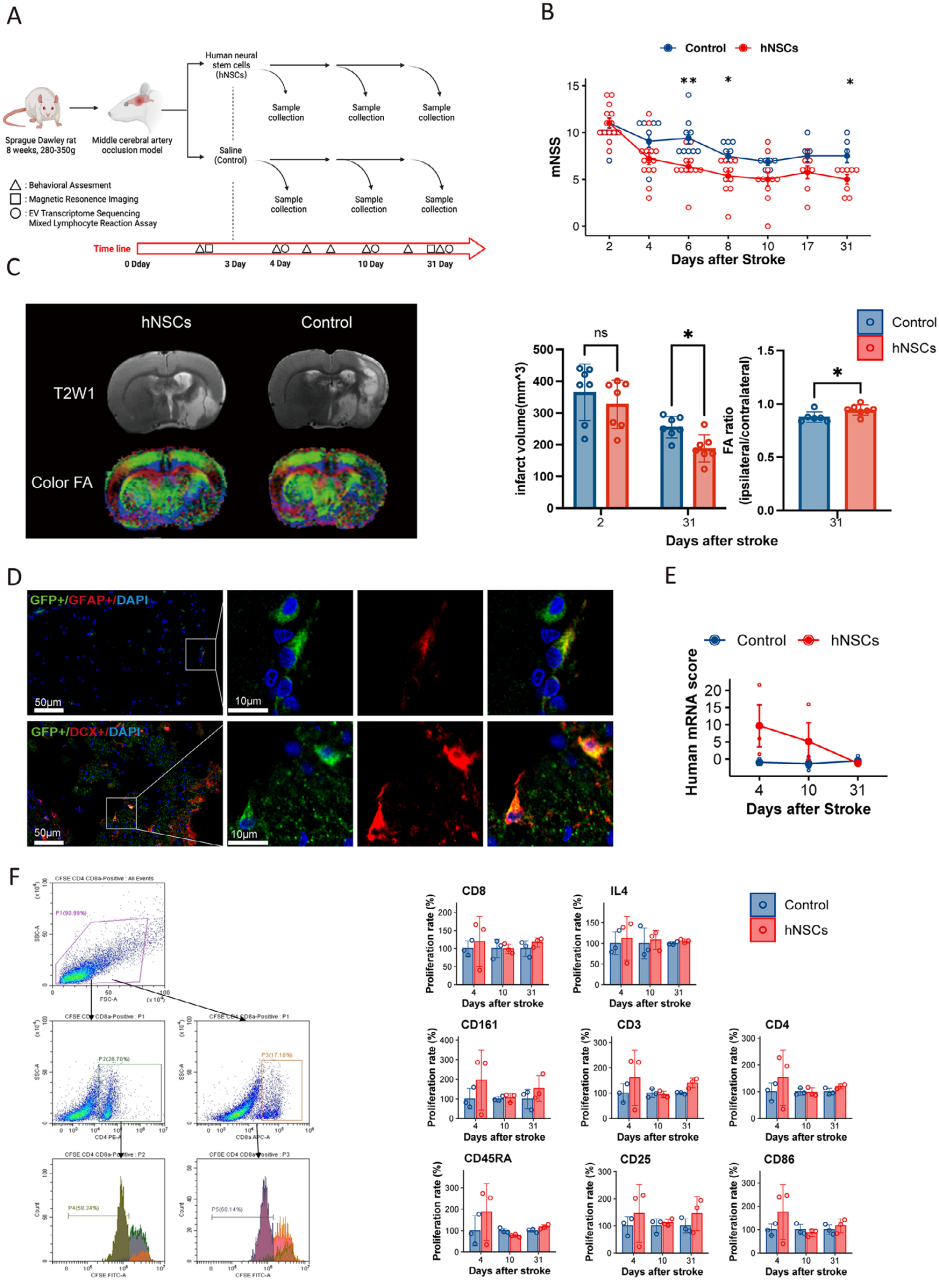
Intra‐arterial delivery of hNSCs enhances functional recovery in stroke‐induced rats. (A) Schematic of the experimental design. (B) Modified neurological severity score (mNSS) evaluation of stroke rats after hNSCs transplantation. *n* = 15 animals per group. Statistical significance was assessed using multivariate analysis of variance. *p < 0.05, **p < 0.01. (C) Left panel: Representative magnetic resonance imaging scans of rat brains with T2‐weighted and diffuse tensor imaging sequences. Middle panel: Quantification of infarct volume in stroke rats after hNSCs transplantation. Right panel: Relative fractional anisotropy in the infarct hemisphere of stroke rats after hNSCs transplantation. *n* = 7 animals per group. Data were compared using Student's *t*‐test. *p < 0.05. (D) GFP^+^ hNSCs differentiated into GFAP^+^ glial cells (upper panel) and DCX^+^ neuroblasts (bottom panel) in the infarcted hemisphere of the brain after transplantation. Scale bar = 50 µm (full view) and 10 µm (magnified view). (E) Quantification of human mRNA detected in rat plasma samples, as measured by *Z*‐scores. *n* = 3 animals per group. (F) Mixed lymphocyte reaction assay on CD3^+^, CD4^+^, CD8^+^, CD161^+^, IL4^+^, CD86^+^, CD45RA^+^, and CD25^+^ splenic cells. Left panel: Representative flow cytometry plots. Right panel: Quantification of the proliferation rate relative to the control group at 4 days post stroke. *n* = 3 animals per group. Data were compared using Student's *t*‐test. *p > *0.05.

hNSCs were dissociated from neurospheres and expressed NSC markers nestin and Sox2 but not immune‐related markers CD86 and HLA‐DR. hNSCs demonstrated their multipotency by differentiating into neuronal lineages expressing β‐III tubulin (Tuj1) and astroglial lineages expressing glial fibrillary acidic protein (GFAP) in vitro (Figure S2A,B). Subsequently, hNSCs (2 × 10^5^ cells per rat in 0.2 mL suspension) or saline (0.2 mL) were administered to stroke‐induced rats via carotid artery injection. Functional tests, brain imaging, and tissue collection were completed within the following month (Figure 3A).

The hNSCs‐treated group showed significant functional recovery compared with the control group (*p *< 0.05 at 6‐, 8‐, and 31‐days poststroke) as measured by the modified neurological severity score (mNSS) (Figure 3B), particularly in motor function recovery (Figure S2C). The adhesive removal test, which assesses sensorimotor function on the affected forelimb, showed an improving trend in the hNSCs‐treated group, although this difference was not statistically significant (Figure S2C). At 31 days, T2‐weighted magnetic resonance imaging (MRI) revealed a significant reduction in ischemic lesion volume in the hNSCs treatment group compared with controls (Figure 3C). Furthermore, diffusion tensor imaging (DTI) demonstrated notable increases in relative fractional anisotropy of white matter tracts in the hNSCs group (Figure 3C). Additionally, there were significant improvements in the relative mean diffusivity (MD) and radial diffusivity (RD) within the hNSCs groups (Figure S2D).

To evaluate the effectiveness of hNSCs transplantation, green fluorescent protein (GFP)‐labeled hNSCs (GFP‐hNSCs) were delivered intra‐arterially. One week posttransplantation, GFP‐hNSCs expressing doublecortin (DCX^+^) neuroblastic or GFAP^+^ glial markers were mainly found within and adjacent to blood vessels in the ipsilateral ischemic hemisphere (Figure 3D). To assess for hNSCs‐secreted EVs in transplanted rats, we assayed for human‐specific mRNAs in plasma EVs. Human‐specific mRNAs were detected in rats transplanted with hNSCs at both 4 and 10 days poststroke but not in nontransplanted rats (Figure 3E).

A mixed lymphocyte reaction assay was conducted to evaluate graft‐host responses elicited by hNSCs transplantation. The assay revealed no significant elevation in the proliferation of lymphocytes (CD3, CD4, CD8, CD45RA), natural killer cells (CD161), splenic dendritic cells (CD25), mast cells (IL‐4), or antigen‐presenting cells (CD86) in the hNSCs group compared with controls (Figure 3F).

Previous studies have reported that NSCs can suppress the inflammatory response induced by stroke [12]. To further investigate the role of NSCs in modulating inflammation within our experimental system, we measured inflammation‐related factors in plasma samples 10 days following stroke‐induced surgery. Our results revealed that none of the cytokines detected via the multiplex cytokine assay showed significant differences between the hNSCs group and the sham control. However, we observed a downward trend in proinflammatory factors such as IL‐1, IL‐2, CCL2, CCL3, CCL20, M‐CSF, and GM‐CSF in the hNSCs group, while IL‐13, an anti‐inflammatory factor, exhibited an upward trend (Figure S2E).

### 2.4 Alterations in EV mRNAs after hNSCs Transplantation

**FIGURE 4:**
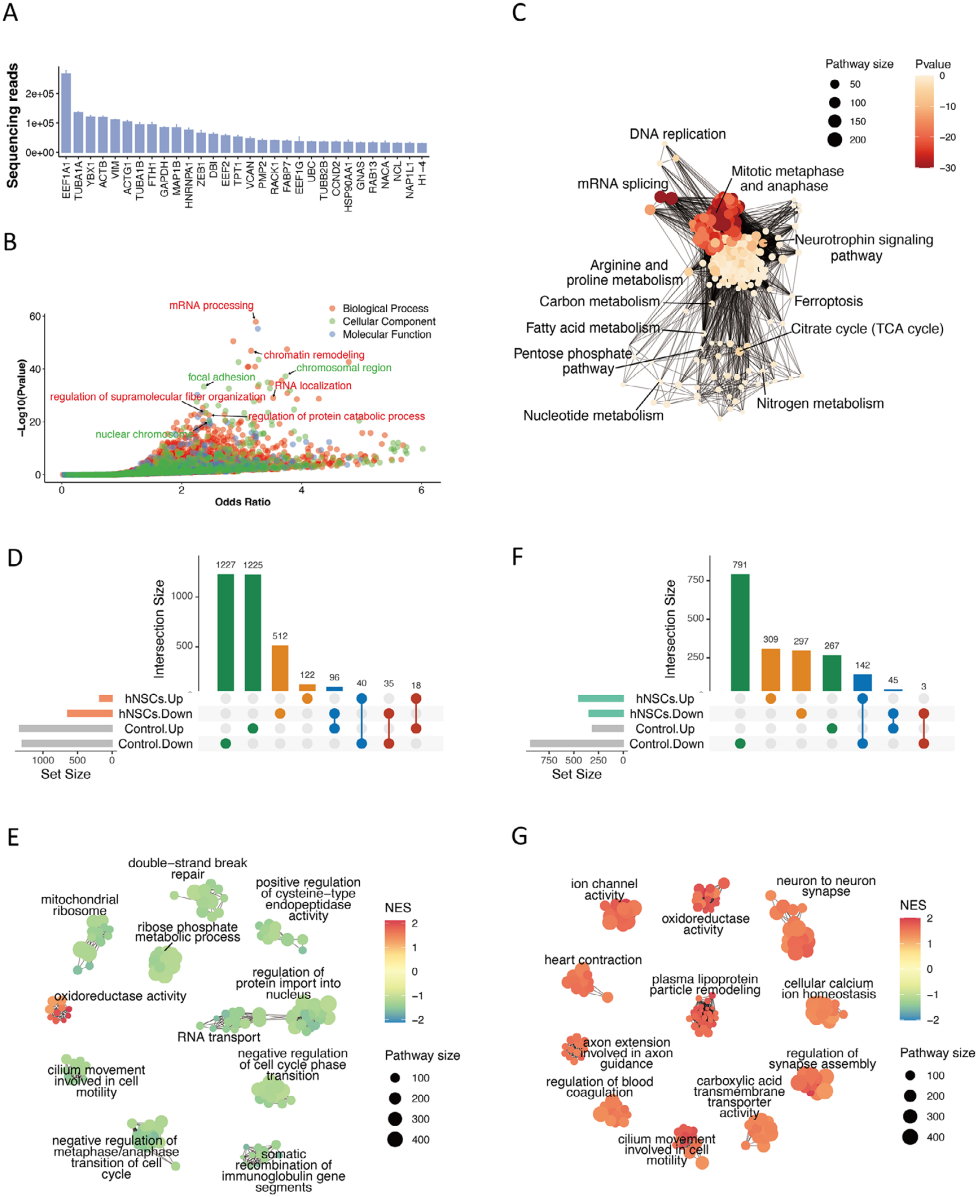
hNSCs‐secreted EV mRNA composition. (A) The top 30 most abundant hNSCs‐secreted EV mRNA cargoes. (B) Gene ontology enrichment dot plot of the top 3000 expressed mRNAs in the hNSCs‐secreted EVs. (C) Network visualization of pathway enrichment analysis for hNSCs‐secreted EV mRNAs. Each dot represents a pathway, while the lines between pathways resemble their shared pathway members. (D) An upset plot visualizing the overlapping patterns of differentially expressed mRNAs detected in brain‐derived EVs. The sets of mRNAs were either upregulated or downregulated in two main comparisons: between hNSCs transplantation and the control group (hNSCs.Up/Down) and between the control and healthy group (Control.Up/Down). (E) Geneset enrichment analysis of the mRNA expression changes between hNSCs transplantation and the control group in brain‐derived EVs. (F) An upset plot visualizing the overlapping patterns of differentially expressed mRNAs detected in plasma‐derived EVs. (G) Geneset enrichment analysis of differentially expressed mRNAs between the hNSCs and the control groups in plasma‐derived EVs. NES refers to the normalized enrichment score, with higher NES representing activation of a pathway.

We isolated EVs secreted by hNSCs from serum‐free culture medium (Figure S3A–C). These EVs were enriched for mRNAs associated with hNSCs differentiation such as elongation factor 1α1 (EEF1A1) and tubulin α1 (TUBA1A) [19]. Gene ontology enrichment analysis of these mRNAs revealed that they participated in chromatin remodeling and mRNA processing (Figure 4A,B). We further analyzed pathway enrichment and grouped pathways with similar gene sets. Hub pathways, including mitotic pathways, neurotrophin signaling, and DNA replication, were identified. Several important metabolic pathways were also observed, including carbon metabolism, fatty acid metabolism, nitrogen metabolism, and nucleotide metabolism. Energy‐associated metabolic pathways, including the citrate cycle and pentose phosphate pathway, were also enriched (Figure 4C and Table S4).

To examine the influence of hNSCs transplantation on rat EV RNA composition, we compared changes in expression between the control group at 4 days poststroke and the healthy group, as well as the expression changes between the hNSCs and control group at 4 days poststroke (Figure S3D,E). There were negative correlations in differentially expressed mRNAs between the stroke control and the hNSCs treatment group in both brain (Pearson *r* = −0.289) and plasma (Pearson *r* = −0.493). Specifically, 96 mRNAs upregulated in brain‐derived EVs at 4 days poststroke were suppressed after hNSCs transplantation (Figure 4D). Gene set enrichment analysis (GSEA) revealed that DNA repair mechanisms and mitochondria‐associated molecules were attenuated after transplantation, while oxidoreductase activity was increased (Figure 4E). In plasma‐derived EVs, 142 mRNAs that were downregulated after stroke at 4 days were elevated in the hNSCs treatment group (Figure 4F). GSEA of plasma‐derived EVs demonstrated that hNSCs transplantation carried neuronal components into the rat circulation and increased ion channel, transmembrane transporter, and oxidoreductase activities (Figure 4G).

### 2.5 Alterations in EV miRNAs after hNSCs Transplantation

**FIGURE 5:**
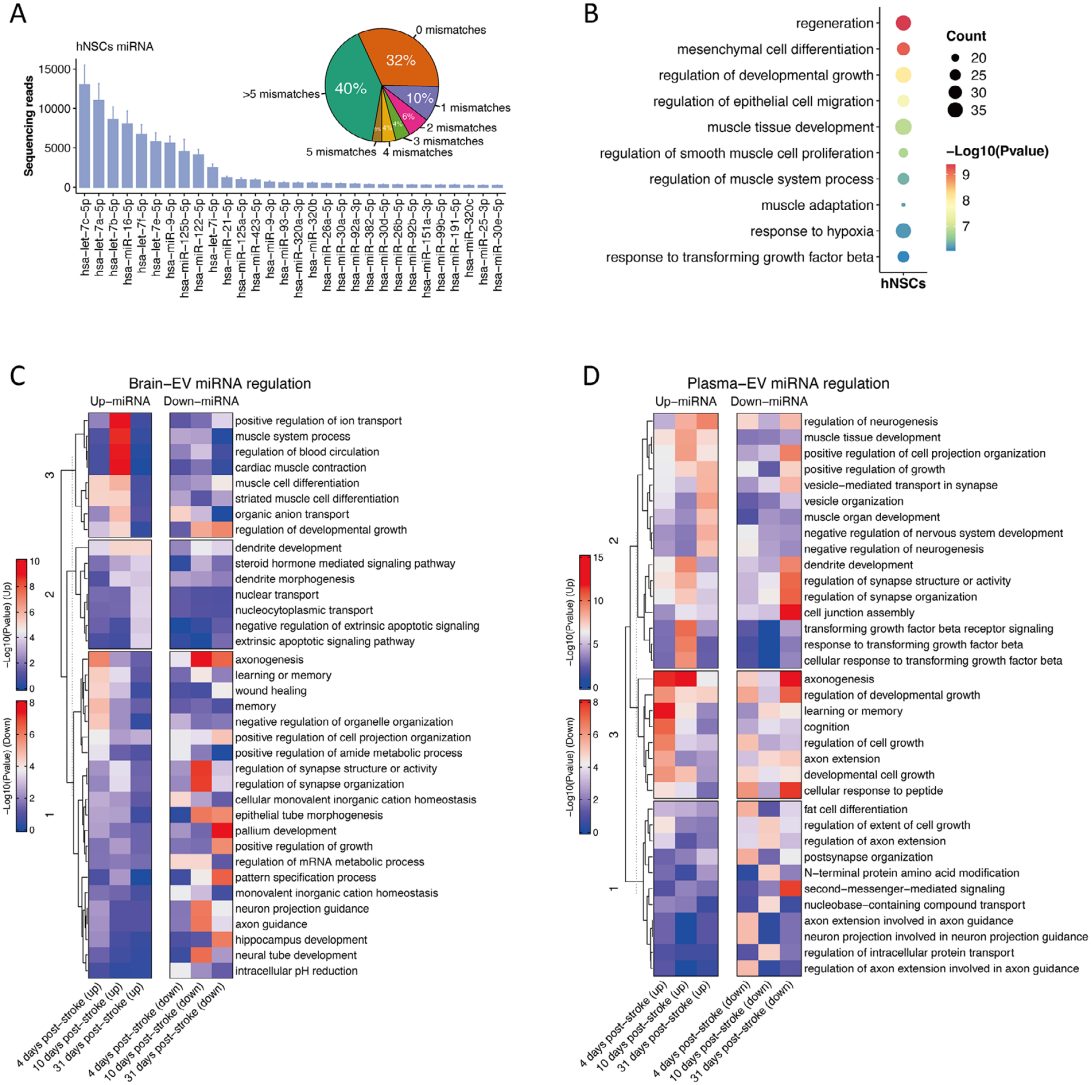
hNSCs‐secreted EV miRNA composition. (A) The top 30 most abundant hNSCs‐secreted EV miRNA cargoes. The pie chart in the upper‐right hand corner shows the proportion of miRNA sequence similarity between *Homo sapiens* and *Rattus norvegicus*. (B) A bubble plot of the top 10 pathways enriched by hNSCs‐secreted EV miRNA‐targeted genes. The size of each circle corresponds to the number of targeted genes within a particular pathway. (C) Heatmaps showing the pathways enriched by the up‐ or downregulated miRNAs in plasma‐derived EVs after hNSCs transplantation. (D) Heatmaps showing the pathways enriched by the up‐ or downregulated miRNAs in brain‐derived EVs after hNSCs transplantation.

There is extensive evidence that EVs secreted by hNSCs exert their therapeutic effect through miRNAs [13]. We found that members of the let‐7 family, miR‐16‐5p, miR‐9‐5p, and miR‐125b‐5p were enriched in hNSCs‐secreted EVs (Figure 5A). miRNAs derived from rats and those secreted by hNSCs have high sequence similarity. Among the 503 miRNAs identified in hNSCs‐derived EVs, 162 (32%) exhibited identical sequences to those in rat miRNAs (Figure 5A and Table S5). About half of these miRNAs possessed sequences with less than three mismatches. We conducted gene enrichment analysis focusing on the target genes of the top 100 most abundant conserved EV miRNAs secreted by hNSCs. These miRNAs regulated biological processes of regeneration, mesenchymal cell differentiation, and epithelial cell migration (Figure 5B and Table S6).

Next, we compared temporal changes in miRNA profiles from brain and plasma EVs between stroke rats receiving hNSCs transplantation and nontransplanted controls at various poststroke time points (Figure S4A,B). In plasma‐derived EVs, upregulated miRNAs at 4 days were involved in axonogenesis, cell growth, and mechanisms associated with learning or memory. At 10 and 31 days after stroke onset, upregulated miRNAs were involved in neurogenesis, dendrite development, synapse organization, and response to transforming growth factor signaling. Downregulated miRNAs in plasma EVs were associated with cell junction assembly, fat cell differentiation, peptide responses, and second‐messenger signaling (Figure 5C and Table S7). In brain‐derived EVs, the upregulated miRNAs were related to wound healing processes at 4 days and ion transport regulation at 10 days. Interestingly, at 10 and 31 days after stroke onset, the enriched downregulated miRNAs were involved in axonogenesis, neuron projection guidance, synapse structure organization, and pallium development (Figure 5D and Table S8).

Huang et al. [20] applied mesenchymal stromal cell transplantation to treat stroke rat models, and isolated EVs from blood plasma samples for miRNA sequencing. They found 61 miRNAs that were differentially expressed with predict targets. We compare their results with our differentially expressed miRNAs derived from plasma EV samples and found that 28% (17 out of 61) of these miRNAs overlapped with our results, including miR‐17‐5p, miR‐20a‐5p, miR‐9a‐5p, miR‐760‐3p, and miR‐125b‐2‐3p (Figure S4C). This demonstrates that there is a significant overlap in the miRNA profiles between the two studies, suggesting a conserved regulatory mechanism in response to stroke and cell‐based therapies. However, the remaining nonoverlapping miRNAs may reflect differences in the therapeutic mechanisms of mesenchymal stromal cells versus hNSCs, as well as variations in experimental design or sample collection timelines.

### 2.6 hNSCs Transplantation Reduces Brain–Plasma Correlations Induced by Stroke

**FIGURE 6:**
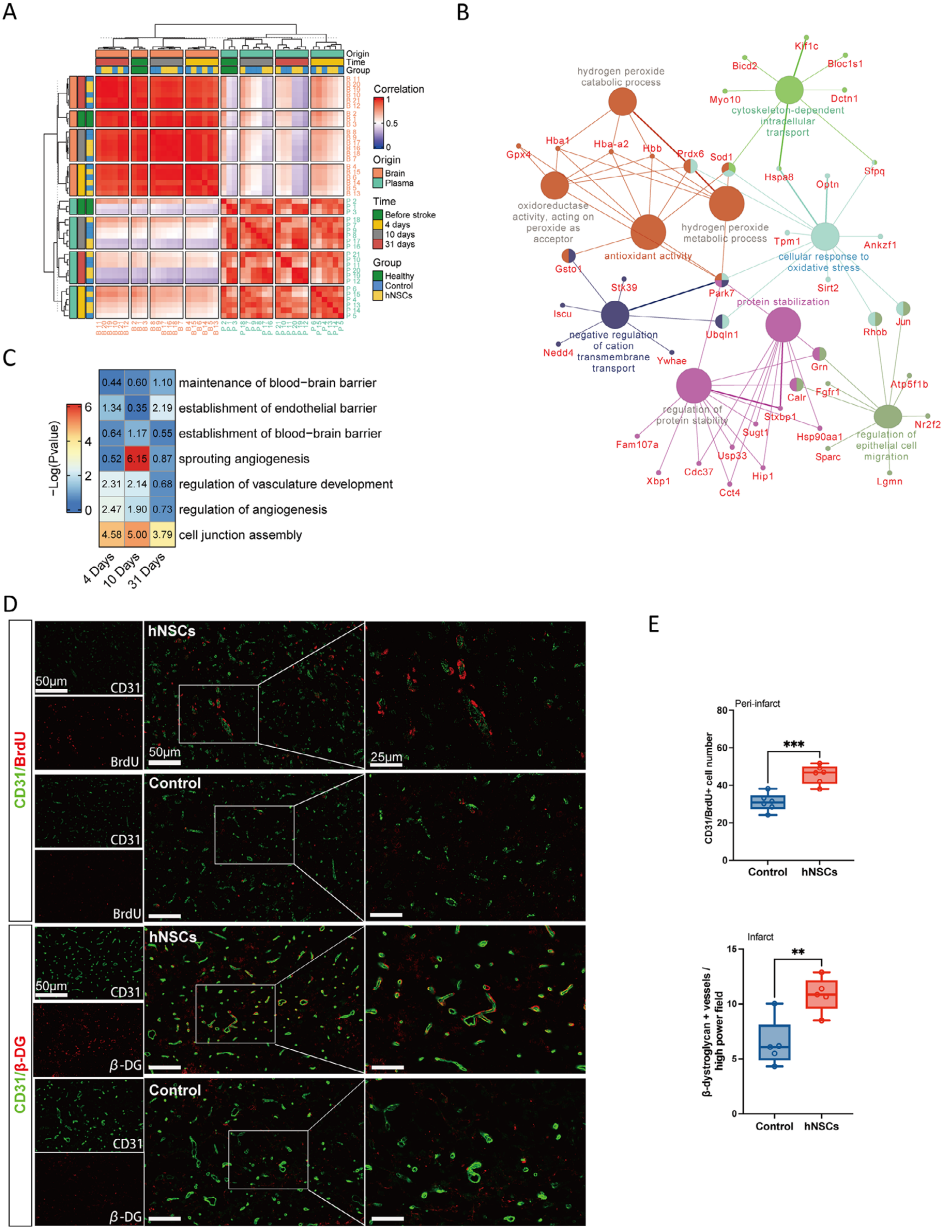
Brain–plasma EV RNA correlation and regulation on blood–brain barrier integrity. (A) Pearson correlations between brain and plasma EV mRNAs visualized in a heatmap. The heatmap was segmented into different sectors representing their origins and time points of collection. (B) Pathway enrichment network of highly correlated mRNAs. Different colors represent different pathway modalities. (C) Pathways related to angiogenesis and blood–brain barrier integrity enriched by differentially expressed brain EV miRNAs after hNSCs transplantation. (D) CD31^+^/BrdU^+^ proliferating endothelial cells in the peri‐infarct areas at 14 days poststroke and βDG^+^ astrocytic end‐feet in the infarct areas at 31 days poststroke. Scale bar = 50 µm (full view) and 25 µm (magnified view). (E) Quantitative assessment of endothelial cell density and astrocytic end‐feet coverage between the two groups within the infarct areas. *n* = 5 rats per group, and each dot in the plot represents an animal assessed. Statistical significance was determined by the Student's *t*‐tests, **p < 0.01, ***p < 0.001.

It has been hypothesized that brain‐derived EVs carry inflammatory signals following acute stroke and transmit them to the peripheral plasma [21, 22]. We performed a joint brain–plasma EV RNA analysis to test this hypothesis. Principal component analysis revealed that EV contents clustered according to tissue of origin (Figure S5A). Notably, correlations between brain and plasma were higher during an acute stroke at 4 days. By contrast, the correlation between brain and plasma was less pronounced without stroke and in those animals receiving hNSCs transplantation at 10 and 31 days poststroke (Figure 6A). Heatmaps visualizing the top 50 most variable mRNAs revealed that brain‐derived EVs exhibited a rich repertoire of markers associated with brain damage including myelin basic protein, S100 calcium‐binding protein B, vimentin, and apolipoprotein E [23, 24]. Importantly, the expression of these molecules was attenuated in the hNSCs transplantation group. Conversely, neuronal markers, including synaptosome‐associated protein 25 kDa, neurogranin, and calcium/calmodulin‐dependent protein kinase II inhibitor 1, were upregulated in the hNSCs group 31 days poststroke. The mRNAs identified in brain‐derived EVs following stroke were also detectable in plasma EVs (Figure S5B). Comparing the brain–plasma correlation between hNSCs and control groups revealed that the control group had more mRNAs with a high correlation (Figure S5C). miR‐21‐5p, miR‐124‐3p, miR‐9a‐5p, and miR‐128‐3p are enriched in the brain and have been reported to have diagnostic value in early‐stage acute stroke [15, 25, 26]. However, only miR‐21‐5p and miR‐124‐3p had high brain–plasma EV correlations in the control group. No significant correlation was found in the hNSCs group (Figure S5D). These results suggest that the correlation between brain and plasma EVs was linked to the acute phase of stroke and may attenuate over time or with hNSCs treatment.

To evaluate the molecular information delivered by EVs during stroke, we performed enrichment analysis based on highly correlated mRNAs in brain and plasma. Associated mRNAs participated in oxidative stress, antioxidant activity, protein stabilization, epithelial cell migration, and transportation mechanisms (Figure 6B). The hub genes connecting these processes included parkinsonism‐associated deglycase (Park7), peroxiredoxin 6 (Prdx6), Jun proto‐oncogene (Jun), and calreticulin. These molecules were highly correlated between brain and plasma, especially in the control groups (Figure S6A). Interestingly, most of them were highly expressed after stroke and downregulated after hNSCs transplantation, both in the brain and plasma (Figure S6B).

### 2.7 Effects of hNSCs on Angiogenesis and the Blood–Brain Barrier

One possible explanation for the reduced brain–plasma correlation after hNSCs transplantation is altered permeability between the CNS and the periphery, with the blood–brain barrier playing a key role in regulating the exchange between the two. We hence explored the possible brain‐derived EV miRNA regulatory mechanisms associated with angiogenesis and blood–brain barrier integrity, finding that angiogenesis was the first process to be regulated by EV miRNAs, followed by the establishment of endothelium and the blood–brain barrier (Figure 6C). Immunofluorescence staining at 14 days poststroke demonstrated enhanced neovascularization in the hNSCs group, evidenced by the presence of CD31^+^/bromodeoxyuridine (BrdU^+^) staining in the peri‐infarct regions (Figure 6D,E). Costaining with β‐dystroglycan (βDG) in astrocytic end‐feet and CD31 in endothelial cells was conducted to further assess the contribution of astrocytes to blood–brain barrier establishment. Notably, at 31 days, the hNSCs group exhibited a significantly expanded CD31^+^/βDG^+^ area within the stroke cavity compared with controls (Figure 6D,E). This observation suggested potentiation of astrocyte end‐feet coverage around vessels and more robust blood–brain barrier integrity following hNSCs treatment.

### 2.8 Effects of hNSCs on Neurogenesis and Neuroprotection

**FIGURE 7:**
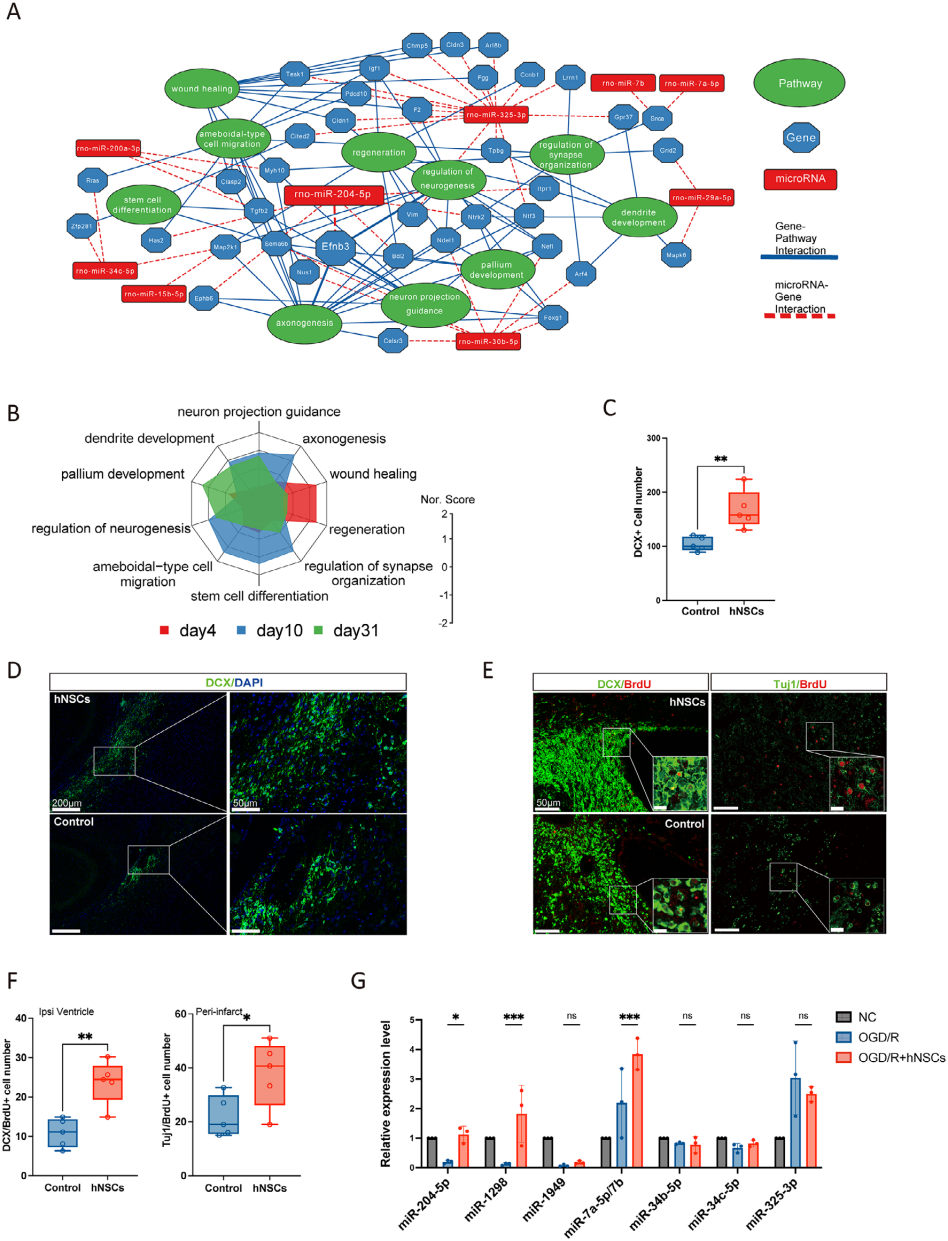
EV miRNAs regulate endogenous neurogenesis and wound healing. (A) Network visualization of the regulatory roles of EV miRNAs and their targeted genes and pathways. Nodes represent pathways, genes, or miRNAs. Lines connecting nodes indicate miRNA–gene interactions or gene‐pathway associations. (B) Radar chart comparing the representative pathways across different time points. Normalized scores (Nor. Score) were calculated by normalizing the log‐transformed *p* value of each pathway. (C) Quantitative analysis of DCX+ neuroblast migration in corpus collosum between the hNSCs and control groups. (D) Representative fluorescence images of migrating neuroblasts in corpus callosum. Scale bar = 200 µm (full view) and 50 µm (magnified view). (E) DCX^+^/BrdU^+^ proliferating neuroblasts in the SVZ and Tuj1^+^/BrdU^+^ proliferating immature neurons in the peri‐infarct regions. Scale bar = 50 µm (full view) and 10 µm (magnified view). (F) Quantitative assessment of DCX^+^ and Tuj1^+^ neurons in the hNSCs and control groups. *n* = 5 animals per group, and each dot in the plot represents each animal assessed. (G) Relative expression of the top miRNAs in HT22 cells after OGD/R. The majority of them also play a role in regulating neuronal processes, according to regulatory network analysis. Of the selected miRNAs, miR‐653 and miR‐431 were not expressed in the qPCR results; miR‐7a‐5p and miR‐7b had similar expression level. *n* = 3 biological replicates per group. Statistical significance was determined by the Student's *t*‐tests, *p < 0.05, ***p < 0.001.

Next, we conducted regulatory network analysis to identify key EV miRNA implicated in stroke recovery (Figure 7A). EV miRNA regulatory network showed that increases in miR‐325‐3p and miR‐204‐5p and decreases in miR‐30b‐5p played pivotal roles in regulating neuronal processes. A comparative analysis of representative regulatory processes at the three poststroke time points revealed that wound healing and regeneration were primarily regulated at 4 days. Axonogenesis, synapse organization, stem cell differentiation, and cell migration were enriched at 10 days. Dendrite development and neuron projection guidance were critical at both 10 and 31 days (Figure 7B).

To validate the results of regulatory network analysis, immunohistochemical analysis was performed on brain tissue harvested 14 days after stroke surgery. In the hNSCs group, there was a significant increase in the migration of DCX^+^ neurons, specifically along the corpus callosum migratory route toward the lesion site (Figure 7C,D). Quantitative analysis of DCX^+^/BrdU^+^ proliferating neuroblasts within the subventricular zone (SVZ) revealed a substantial increase in cellular proliferation in the hNSCs group compared with control animals. Furthermore, neuronal differentiation, assayed via Tuj1^+^/BrdU^+^ double‐staining, was significantly upregulated in the peri‐infarct regions of brains treated with hNSCs, validating the neurogenesis and migratory benefits associated with hNSCs transplantation (Figure 7E,F). Additionally, at 31 days poststroke, reduced glial scar formation was evident in the peri‐infarct regions of the hNSCs‐treated group, as demonstrated by GFAP staining. Notably, a decrease of ionized calcium‐binding adaptor molecule 1 (Iba‐1^+^) microglia was also observed in the hNSCs group (Figure S6C).

### 2.9 hNSCs Transplantation Confers Neuroprotection Through Upregulating miR‐204‐5p

To investigate the biological effects of differentially expressed miRNAs, we conducted in vitro experiments using the HT22 hippocampal neuronal cell line and choose top 10 upregulated miRNAs in hNSCs group (Table S8), of which majority of them have been implicated in neuronal processes regulatory networks. After coculturing oxygen‐glucose deprivation/reoxygenation (OGD/R) neurons with hNSCs, qPCR analysis revealed that, among the differentially expressed brain‐derived miRNAs, the expression of miR‐1298, miR‐7a‐5p, and miR‐204‐5p was significantly increased (Figure 7G). While the neuroprotective effect miR‐7 has been shown in promoting functional recovery by inhibiting α‐synuclein‐mediated neuronal death [27], elevated miR‐204‐5p levels have been detected in the cerebrospinal fluid of stroke patients [28]. Consequently, our subsequent mechanistic studies focused on the effects of upregulated miR‐204‐5p after hNSCs transplantation.

**FIGURE 8:**
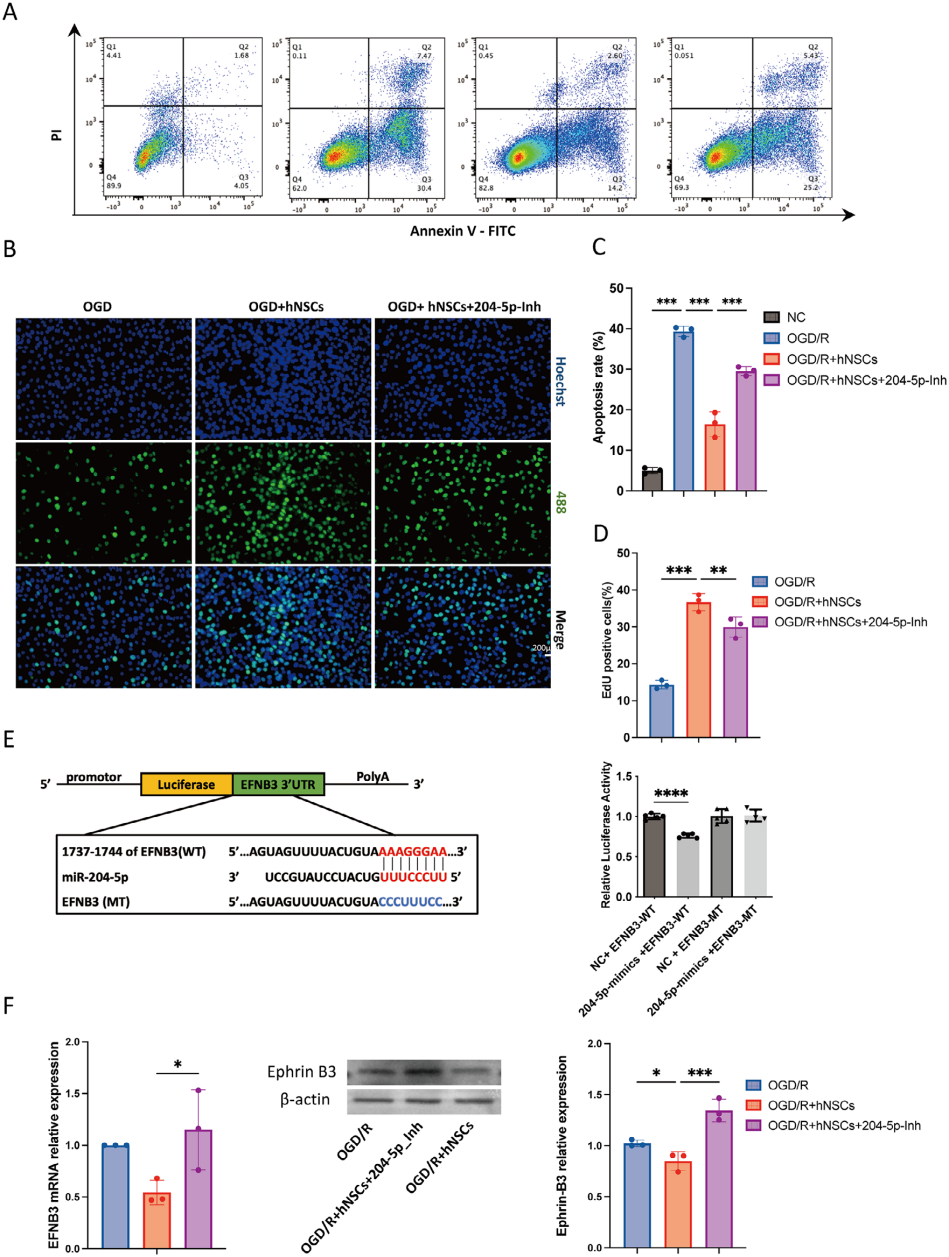
miR‐204‐5p in hNSCs‐mediated neuroprotection against OGD/R. (A) Annexin V‐FITC/PI apoptosis assay assessing apoptotic cell proportions in experimental groups from left to right: normal control (NC), oxygen‐glucose deprivation/reoxygenation (OGD/R), OGD/R cocultured with human neural stem cells (OGD/R+hNSCs), and OGD/R+hNSCs with miR‐204‐5p inhibitor (OGD/R+hNSCs+204‐5p‐Inh). (B) Representative fluorescence images of EdU+/Hoechst‐stained proliferating neuronal cells following OGD/R. EdU (green) labels proliferating cells; nuclei are counterstained with Hoechst (blue). Scale bar = 200 µm. (C) Quantitative analysis of apoptotic cell percentages across groups (Annexin V‐FITC/PI assay). (D) Quantification of proliferating cell percentages across groups. (E) Left panel: Predicted binding sites between miR‐204‐5p and EFNB3 3′‐UTR, with schematic of wild‐type (WT) and mutant (MT) EFNB3 constructs for luciferase assays. Right panel: Relative luciferase activity in cells cotransfected with miR‐204‐5p mimics (or NC mimics) and WT/MT EFNB3 reporter plasmids. (F) Left panel: qPCR analysis of EFNB3 mRNA levels. Middle panel: Representative WB bands for Ephrin B3 and β‐actin. Right panel: WB quantification of Ephrin B3 protein expression. Data are presented as mean ± SD (*n* = 3 biological replicates). Statistical significance was determined by one‐way ANOVA followed by Student's *t*‐tests for pairwise comparisons, *p < 0.05, **p < 0.01, ***p < 0.001, ****p < 0.0001.

We then performed apoptosis assays using Annexin V‐FITC/PI double staining to quantify apoptotic rate for neurons before and after OGD/R induction. A significant increase in apoptosis was identified in OGD/R neurons (Figure 8A,C). Cell apoptosis was reduced when coculturing OGD neurons with hNSCs, while the addition of the miR‐204‐5p inhibitor partially reversed this effect, suggesting that miR‐204‐5p plays a role in promoting neuronal survival (Figure 8C).

We also conducted EdU/Hoechst staining to assess neuronal proliferation. The results demonstrated a significant increase in the number of EdU+ cells after coculturing with hNSCs in the OGD/R model. However, the addition of the miR‐204‐5p inhibitor partially inhibited proliferation (Figure 8B,D). A similar result was observed using flow cytometry, which showed an increased proportion of EdU+ cells in the hNSCs coculture group (Figure S6E). This proportion was reduced upon the addition of the miR‐204‐5p inhibitor, suggesting that miR‐204‐5p plays a role in promoting neuronal proliferation following hNSCs transplantation.

To systematically identify functional target genes of miR‐204‐5p, a joint prediction approach was employed using three major databases: TargetScan [29], miRDB [30], and TarBase [31] (Figure S6D). Integration of the target gene predictions from all three databases revealed an overlap of 27 common genes. Among these candidates, Ephrin‐B3 (EFNB3) was identified as a potential target gene of miR‐204‐5p for further investigation in this study. Ephrins constitute a family of transmembrane or glycosylphosphatidylinositol‐anchored ligands for receptor tyrosine kinases. They are categorized into two subclasses: Ephrin‐A (A1–A5) and Ephrin‐B (B1–B3). Ephrins regulate neural development, axon guidance, and synaptic plasticity through bidirectional signaling with their cognate Eph receptors (EphA/EphB) [32]. Dual‐luciferase reporter assays confirmed that miR‐204‐5p directly binds to EFNB3 and downregulates its luciferase activity (Figure 8E). Additionally, qPCR results revealed that EFNB3 expression was reduced in the hNSCs group, and the expression can be upregulated by the addition of miR‐204‐5p inhibitor. Western blot analysis further confirmed the level of EFNB3 is decreased in the hNSCs group; transfection with the miR‐204‐5p inhibitor significantly increased EFNB3 protein expression (Figure 8F). From the experiments presented, our findings suggest that hNSCs can promote neuronal proliferation and inhibit apoptosis by upregulating miR‐204‐5p, potentially exerting a neuroprotective effect through the regulation of EFNB3.

## 3 Discussion

This study characterized temporospatial transcriptomic signatures of EVs during the pathophysiological progression of stroke and after hNSCs treatment. Our findings revealed that, shortly after stroke onset, neuronal EV secretion decreased in the brain, coinciding with upregulation of EV secretion from microglia, endothelial cells, pericytes, and other nonbrain‐derived cells. In addition, due to the damage of the blood–brain barrier and the release of inflammatory factors from the ischemic region, peripheral immune cells including leukocyte could infiltrate into the brain, as supported by a decrease in the purity of brain‐specific EV RNAs. An increased correlation between brain and plasma EVs also suggests compromised blood–brain barrier integrity. For instance, miR‐34a‐5p and miR‐24‐3p, which were associated with endothelial cells and angiogenesis [33, 34], were upregulated in EVs from both brain and plasma after stroke. This likely reflects their release by brain endothelial cells into peripheral circulation following blood–brain barrier damage. We observed increased angiogenesis and astrocyte end‐feet coverage around brain microvasculature in the hNSCs group. Moreover, the correlation between brain and plasma EV RNAs decreased in the hNSCs‐transplanted group compared with controls, indicating improved blood–brain barrier integrity and basement membrane restoration following hNSCs transplantation.

Intravascular transplantation is an efficient route for delivering hNSCs to treat cerebral infarction. hNSCs express integrin and chemokine ligands and can cross the blood–brain barrier, migrating into the brain parenchyma and localizing to the infarction site [35]. Our findings demonstrate that administrating hNSCs via intra‐arterial injection enables the stem cells to reside in the infarct penumbra adjacent to blood vessels and survive for over 1 week. Notably, while hNSCs‐derived EVs decreased within 1 month postinjection, the resulting functional improvements persisted beyond this period, suggesting that neuronal recovery involves mechanisms beyond cell replacement. Previous studies have proposed that NSCs‐mediated tissue repair may occur primarily through bystander effects rather than direct neuronal integration [36]. By using BrdU to label newborn neurons, we demonstrate that exogenous hNSCs transplantation can also promote endogenous neurogenesis. Furthermore, hNSCs transplantation exerted an inhibitory effect on glial scar formation and microglial activation. These results suggest that the transplanted hNSCs modulate ischemic lesion microenvironment, resulting in a prolonged therapeutic effect.

Following hNSCs transplantation, specific miRNAs, including miR‐204‐5p, miR‐7‐5p, and miR‐325‐3p, were significantly upregulated in brain‐derived EVs. These miRNAs regulate neurogenesis, synapse organization, wound healing, angiogenesis, and blood–brain barrier establishment. To further validate the functional impact of the altered miRNA, we investigated the role of miR‐204‐5p in HT22 neurons coculture with hNSCs in vitro. Consistent with findings from Xiang et al. [37], miR‐204‐5p improved neuron survival under ischemic conditions. Notably, we found that hNSCs enhanced neuronal proliferation, as evidenced by increased EdU+ cell proportion following ischemia, an effect that was attenuated by miR‐204‐5p downregulation. We further identified EFNB3 as the target gene of miR‐204‐5p, and EFNB3 expression is repressed with hNSCs coculture. EFNB3, a transmembrane ligand of the Eph receptor family, plays a dynamic regulatory role in neuronal proliferation and neuroblast migration. Previous studies have shown that EFNB3 negatively regulates endogenous neurogenesis in the adult CNS, while cell proliferation in the SVZ regions is significantly enhanced in EFNB3 knockout mice after ischimic brain injury [38, 39]. Together, our experiments confirm the proneurogenesis effects of hNSCs and propose a potential mechanism through the miR‐204‐5p/EFNB3 axis.

In a clinical context, the molecules present in plasma‐derived EVs have the potential to serve as biomarkers for diagnosing stroke and monitoring responses to treatment. Our study revealed that the expression levels of miR‐542‐3p and miR‐34a‐5p in these plasma‐derived EVs were closely linked to their counterparts originating from the brain and were upregulated following a stroke. This indicates their suitability as biomarkers for stroke diagnosis. Additionally, Park7, Prdx6, and Jun in plasma EVs might serve as markers of pathological processes associated with stroke. However, despite the promising potential of EVs as biomarkers for stroke, their clinical application faces significant practical challenges, particularly in sample quality control. Blood‐derived EV analysis is complicated by the presence of confounding components such as lipoproteins, erythrocyte debris, and nonvesicular nucleic acids, which hinder accurate isolation and interpretation [40]. To mitigate these issues, standardized protocols aligned with International Society for Extracellular Vesicles guidelines must be adopted: (1) anticoagulant tubes (e.g., citrate/EDTA) should be used to suppress platelet activation; (2) processing delays between collection and centrifugation must be minimized to reduce leukocyte/platelet contamination; (3) strict freeze–thaw conditions are essential to preserve EV integrity; (4) hemolysis should be monitored and reported to exclude red blood cell‐derived artifacts; and (5) platelet‐specific markers (e.g., CD41/CD61) may be assessed to quantify contamination [41]. Importantly, EV profiles are highly sensitive to donor status and collection methods, necessitating meticulous documentation and protocol harmonization [42, 43]. Future studies should prioritize multicenter validation with standardized workflows to bridge the gap between experimental findings and real‐world clinical utility.

This study has some limitations. First, the use of young rats, approximately 2–3 months old, does not accurately represent the demographic of stroke patients, who are primarily older individuals. To address this, future in vivo studies will use older rat models and observe them over an extended period to more accurately evaluate the long‐term therapeutic effects of NSCs transplantation. Second, the single‐cell RNA sequencing data utilized for EV source tracking originated from healthy rats, rather than stroke‐induced animals. Consequently, our cell type signature matrix may lack certain disease‐related cell subtypes, potentially underestimating the complexity of the cellular interactions within the stroke microenvironment. To overcome these limitations, future studies will integrate brain single‐cell sequencing from stroke‐induced rats. Another limitation is that while we observed differentially expressed miRNAs in brain‐derived EVs, the exact mechanism by which hNSCs upregulate these miRNAs is still unknown. Future studies will focus on identifying specific pathways through which exogenous hNSCs regulate miRNA expression. In particular, we will investigate the impact of inhibiting hNSCs secretion of EVs on therapeutic outcomes, to evaluate whether the indirect effects of hNSCs are mediated through EVs secretion. Nevertheless, our findings provide valuable insights into the EV miRNA regulatory networks governing stroke pathophysiology and highlight the therapeutic potential of hNSCs and the underlying mechanisms.

## 4 Conclusion

Ischemic stroke, a leading cause of chronic neurological disability with limited therapeutic options, underscores the critical need for innovative treatments and monitoring strategies. Our study demonstrates that EV‐derived RNAs, particularly miRNAs, serve as dynamic biomarkers reflecting stroke pathophysiology and the therapeutic response to hNSCs transplantation. By profiling EV‐derived RNAs in rat brain and plasma, we established their cellular origins and documented significant alterations following stroke and after intra‐arterial hNSC administration. Crucially, we identified EV‐derived miRNAs as key mediators of stroke recovery, regulating neurogenesis, angiogenesis, and endothelial barrier integrity. Among these, miR‐204‐5p emerged as a potent regulator of neurogenesis through its targeting of EFNB3. These findings collectively highlight the dual potential of EV RNAs: as noninvasive biomarkers for tracking cell therapy efficacy and as therapeutic targets to augment neural repair after ischemic stroke.

## 5 Methods

Comprehensive materials and methods can be found in the Supporting Information.

### 5.1 hNSCs Isolation

Primary hNSCs was obtained from an hNSCs line derived from human fetus telencephalon at Beijing Yinfeng Dingcheng Biological Engineering Technology Co Ltd (Beijing, China) [44]. The ethics committees of Peking Union Medical College Hospital (approval no. 2021‐01) approved the acquisition of hNSCs, and the quality of isolated hNSCs was verified by the National Medical Products Administration of China (certificate no. SH202001141).

### 5.2 Animal Experiments

Forty adult male Sprague–Dawley rats, aged 8–12 weeks, were subjected to transient MCAO in the right hemisphere as described previously [45]. Briefly, the external carotid artery was transected at its distal end, and a nylon thread occluder coated with silicon was inserted into the bifurcation point of the common carotid artery and internal carotid artery to occlude the middle cerebral artery. After 90 min of occlusion, rats were reanesthetized and the occluder withdrawn to facilitate reperfusion. On the third day poststroke surgery, the common carotid artery was surgically re‐exposed and injected with either a suspension of 2 × 10^5^ hNSCs or saline using a 31‐gauge needle. The rats were distributed evenly into the hNSCs and control groups, ensuring that each group contained individuals with similar baseline behavioral assessment scores.

Behavior of stroke rats was evaluated longitudinally using the mNSS and adhesive removal test [46]. For the imaging cohort, GFP‐labeled hNSCs was injected using identical techniques. To assess cell proliferation, BrdU was administered intraperitoneally at 50 mg/kg for 3 consecutive days before euthanasia. The Experimental Animal Welfare Ethics Committee of Peking Union Medical College Hospital approved all animal procedures (XHDW‐2023‐0320), ensuring ethical compliance and animal welfare.

### 5.3 Mixed Lymphocyte Reaction Assay

Spleen tissue from three animals per group was gently homogenized, and the harvested cells were labeled using a CFSE Cell Division Tracker Kit. Cells were plated at a defined ratio of 3 × 10^5^ immune cells to 1 ×  10^5^ neuronal cells and incubated at 37°C with 5% CO_2_ for 5 days. Following cultivation, cells were collected and immunostained with APC anti‐rat CD3, PE anti‐rat CD4, APC anti‐rat CD8a, APC anti‐rat CD25, APC anti‐rat CD45RA, PE anti‐rat CD86, PE anti‐rat CD161, and PE anti‐rat IL‐4 antibodies, respectively, and prepared for flow cytometry analysis.

### 5.4 Multiplex Cytokine Assay

Supernatants were collected from lysed from rat serum samples using a RIPA lysis buffer. For each sample, 50 µL of supernatant was incubated with antibody‐coupled fluorescent beads. After incubation, the plates were washed with wash buffer to remove unbound proteins. Biotinylated detection antibodies specific to each cytokine were added and incubated at room temperature. Streptavidin–phycoerythrin conjugate was added to each well and incubated. Samples were analyzed using the Luminex‐200 flow‐based system (Luminex Corporation, Austin, TX, USA).

### 5.5 Magnetic Resonance Imaging

T2‐weighted MRI and DTI were acquired 2 and 31 days after stroke onset. Seven animals per group were used for MRI analysis. The T2‐weighted images, processed with ITK‐SNAP software, were utilized to quantify the infarct volume in rats that underwent stroke surgery. The DTI data underwent preprocessing utilizing MRIcroGL, FSL's DTIFIT tool, and DSI studio.

### 5.6 Immunofluorescence Staining and Quantification

Cryosectioning was performed at 30 mm using a microtome for brain sections. Sections were stained with the primary antibodies including anti‐nestin, anti‐GFAP, anti‐CD31, anti‐DCX, anti‐Tuj1, anti‐Iba‐1, anti‐βDG, BrdU, and anti‐GFP overnight at 4°C. Primary antibodies were detected using appropriate secondary antibodies. Sections were subsequently stained with 4′, 6‐diamidino‐2‐phenylindole and visualized using a laser scanning confocal microscope.

### 5.7 EV Isolation and Validation

Plasma EVs and hNSCs‐secreted EVs were isolated from culture supernatants using size exclusion chromatography techniques described previously [47]. EVs were isolated from brain tissue using a protocol modified from Vella et al. [48]. We used the ipsilateral (right) hemisphere of stroke model brains, which were immediately frozen in liquid nitrogen and stored at −80°C until processing. Extracted EVs were validated by nanoparticle tracking analysis, transmission electron microscopy, and western blot analysis of characteristic EV surface proteins.

### 5.8 RNA Isolation and Sequencing from EVs

Total RNA was extracted and purified from EVs utilizing the miRNeasy Advanced Kit. The SMARTer Stranded Total RNA‐Seq Kit and QIAseq miRNA Library Kit were used to construct mRNA and miRNA libraries. Library quality was assessed using the Fragment Analyzer Qseq100 and qPCR. Libraries were sequenced on the Illumina NovaSeq 6000 platform, generating paired‐end reads.

### 5.9 Transcsriptome Analyses

Raw data in FASTQ format were first processed using in‐house Perl scripts. The R package *limma* was used to identify differentially expressed mRNAs and miRNAs [49]. The R package multiMiR was used to identify miRNA target genes [50], and clusterProfiler was used for gene enrichment analysis [51]. The R package *GSVA* was used with default settings to calculate Z‐scores [52]. *Mfuzz* was used for gene coexpression detection [53]. aPEAR, a pathway clustering method, was applied to identify representative enrichment terms [54]. We utilized TissueEnrich for TSG enrichment analysis [55] using GSE53960 to identify rat TSGs [56]. To determine the cellular origin of brain‐derived EVs, we employed scMappR for EV source tracking [57]. This approach utilizes an RNA‐seq deconvolution method, which mathematically estimates the constituent components of a mixture by analyzing observed signals against a predefined signature matrix. The signature matrix consists of gene expression profiles from pure cell types, derived from reference single‐cell RNA‐seq data (GSE250245) [58]. This dataset comprises single‐cell RNA‐seq samples from the right hemispheres of male rodents undergoing sham surgery, moderate ipsilateral MCAO, or severe ipsilateral MCAO.

### 5.10 Cell Culture, OGD/R Induction, and Transfection

HT22 hippocampal neuronal cells were maintained in high‐glucose Dulbecco's modified Eagle medium supplemented with fetal bovine serum and penicillin–streptomycin at 37°C under 5% CO_2_. For OGD/R modeling: cells were washed twice with PBS, and the medium was replaced with glucose‐free DMEM. Cultures were transferred to an anaerobic chamber infused with a gas mixture of 2% O_2_, 5% CO_2_, and 93% N_2_ for 6 h; after OGD, glucose‐free medium was replaced with standard high‐glucose DMEM, and cells were returned to a normoxic incubator for 24 h of recovery. For in vitro coculture: hNSCs was seeded into transwell inserts at a density of 5 × 10^4^ cells/insert and positioned above the HT22 monolayer for another 24 h. In cell transfection experiments, HT22 cells were transfected with either a miR‐204‐5p inhibitor or negative control using Lipofectamine 3000 immediately after reoxygenation. Cells were harvested 48 h posttransfection for further analysis.

### 5.11 Cell Apoptosis and Proliferation Assays

For apoptosis assessment, HT22 cells were harvested, resuspended in binding buffer (1 × 10⁶ cells/mL), stained with 5 µL Annexin V‐FITC and 5 µL PI, and incubated for 15 min in the dark. Apoptotic populations—viable (Annexin V^−^/PI^−^), early apoptotic (Annexin V⁺/PI^−^), late apoptotic (Annexin V⁺/PI⁺), and necrotic (Annexin V^−^/PI—were quantified using an Attune NxT flow cytometer and analyzed with FlowJo. For proliferation analysis, cells from each experimental group were incubated with EdU for 1 h at 37°C to evaluate proliferation rates, using the EdU Cell Proliferation Kit with Alexa Fluor 488. Fluorescence images were captured using a Nikon Eclipse fluorescence microscope. The number of EdU‐positive cells (green) relative to the total nuclei (blue) was quantified from three randomly selected fields using ImageJ software. For flow cytometry, EdU‐labeled cells were analyzed using Attune NxT flow cytometer and FlowJo software.

### 5.12 Dual Luciferase Assay

The predicted binding site of miR‐204‐5p in the EFNB3 3′‐UTR was cloned and inserted into a pmirGLO vector to create the EFNB3 WT construct. The corresponding mutant, EFNB3 MT, was generated by site‐directed mutagenesis. HEK 293 cells were transfected with pmirGLO vectors and miR‐204‐5p mimic/negative control using Lipofectamine 3000. Forty‐eight hour posttransfection, firefly and Renilla luciferase activities were quantified using the using the Dual‐Luciferase Reporter Gene Assay Kit. Data are expressed as relative luciferase activity (Firefly/Renilla).

### 5.13 RT‐qPCR

Total RNA was extracted using TRIzol reagent and was then reverse transribed into cDNA using an all‐in‐one cDNA synthesis supermix and a miRNA first‐strand cDNA synthesis reagent kit by tailing A. mRNA and miRNA expression levels were quantified using SYBR Green Master Mix. Relative expression levels were normalized to U6, which served as the internal reference. Primer sequences are provided in The Supplementary Material (Table S9).

### 5.14 Statistical Analysis and Data Visualization

Continuous variables are presented as mean ± standard deviation (SD), while analysis of variance (ANOVA) or Student's *t*‐tests were used to determine differences between groups. A *p* value <0.05 was considered statistically significant. To visualize data, heatmap plots were created with ComplexHeatmap [59]; volcano plots, boxplots, and bubble plots with ggplot2 [60]; upset plots using UpSetR [61]; and miRNA–gene network plots with Cytoscape (v3.9.1, https://cytoscape.org/).

## Author Contributions

C.C. and Y.W. performed experiments, analyzed data, and interpreted results. X.L. conducted MRI acquisition process. C.C. and Y.W. wrote the manuscript with input from all coauthors. X.B. and R.W. contributed to critical revisions and approved the final version for submission.

## Conflicts of Interest

The authors declare no conflicts of interest.

## Ethics Statement

The ethics committees of Peking Union Medical College Hospital (approval no. 2021‐01) approved the acquisition of hNSCs, andthe quality of isolated hNSCs was verified by the NationalMedical Products Administration of China (certificate no.SH202001141). The Experimental Animal Welfare Ethics Committee of Peking Union Medical College Hospital approved all animal procedures (XHDW‐2023‐0320), ensuring ethical compliance and animal welfare.

## Supporting information




**Supplementary File 1**: mco270400‐sup‐0001‐SuppMat.docx.

**Supplementary File 2**: mco270400‐sup‐0002‐TableS1.xlsx.

**Supplementary File 3**: mco270400‐sup‐0003‐TableS2.xlsx.

**Supplementary File 4**: mco270400‐sup‐0004‐TableS3.xlsm.

**Supplementary File 5**: mco270400‐sup‐0005‐TableS4.xlsx.

**Supplementary File 6**: mco270400‐sup‐0006‐TableS5.xlsx.

**Supplementary File 7**: mco270400‐sup‐0007‐TableS6.xlsx.

**Supplementary File 8**: mco270400‐sup‐0008‐TableS7.xlsx.

**Supplementary File 8**: mco270400‐sup‐0009‐TableS8.xlsx.

## Data Availability

All raw and processed data related to this study have been deposited in the Gene Expression Omnibus repository via accession number GSE268442. Other data regarding this study are available from the corresponding authors upon reasonable request.
