# Supplementary material for: Molecular Encoding of Ischemic Stroke and its Resolution after Human Neural Stem Cell Therapy by Extracellular Vesicles
Source: MedComm (2020). 2025 Oct 28;6(11):e70400. doi: 10.1002/mco2.70400 (PMC12559856; doi:10.1002/mco2.70400)
Supplement: Supplementary file 1 — mco270400‐sup‐0001‐SuppMat.docx. [file MCO2-6-e70400-s005.docx]

**Supplementary Information**

**Supplementary Figures and Figure Legends**

**Supplementary Figure 1. Rat EV characterization and cargo profiles.**

**A,** Western blot analysis of rat brain-derived EVs. The input in lane 1 was lysed rat brain homogenate, while lane 2 contained EVs extracted from rat brain tissue. Characteristic EV membrane proteins were validated using antibodies targeting TSG101, CD9, and HSP70. Calnexin, specifically expressed in the endoplasmic reticulum, served as the quality control marker. **B,** Transmission electron micrographs of EVs from rat brain tissues (left) and rat plasma (right). Scale bar = 200 nm. **C,** Nanoparticle tracking analysis from rat brain tissue and plasma EVs. The diameter calculation was based on the Brownian motion of different movement speeds. **D,** Clustered heatmap of brain-derived EV genes with the highest degree of conformity to the central trajectories. **E,** The single-cell RNA-seq samples were obtained from the right hemispheres of male rodents subjected to sham surgery (Sham_R), moderate ipsilateral middle cerebral artery occlusion (mMCAO_ipsi), or severe ipsilateral middle cerebral artery occlusion (sMCAO_ipsi). The Uniform Manifold Approximation and Projection (UMAP) plots illustrate the cell clusters, annotated by their origin (surgical group) and major cell types. **F,** The dot plot displays representative classical genes used for major cell type classification. **G,** Clustered heatmap of the plasma-derived EV genes with the highest degree of conformity to the central trajectories. **H,** Correlation plots of the six mRNA with the highest Pearson correlations between the brain and plasma.

**Supplementary Figure 2. hNSC characterization, behavioral assessment, magnetic resonance imaging, and multiplex cytokine assay.**

**A,** Upper panel: Morphology of neurospheres as visualized by light microscopy. Scale bar = 200 μm. Middle and lower panels: Immunofluorescent staining of *in vitro*-cultured hNSCs differentiated into Tuj1^+^ immature neurons and Gfap^+^ glial cells. Scale bar = 100 μm. **B,** Flow cytometry analysis targeting hNSC membrane molecules nestin, Sox2, HLA-DR, and CD86. hNSCs were labeled as green or red lines, with or without antibody staining. **C,** The series of tests in the mNSS including motor, balance and sensory-reflex (n=15 per group). Adhesive removal test measuring sensorimotor functions (n=5 per group). Statistical significance was assessed using multivariate analysis of variance. **P*<0.05, ***P*<0.01, ****P*<0.001. **D,** Upper panel: representative diffuse tensor imaging (DTI). The metrics provided by DTI include axial diffusivity (AD), mean diffusivity (MD), and radial diffusivity (RD). Bottom panel: quantitative results of DTI metrics. n=6 animals in the control group and n=7 in the hNSC group. Statistical significance was assessed using Student's *t*-test. **P*<0.05. **E,** Multiplex cytokine assay measuring cytokines in rat serum 10 days post-stroke.

**Supplementary Figure 3. hNSCs EV characterization and differentially expressed mRNAs after hNSC transplantation.**

**A,** Western blot analysis of hNSCs-secreted EVs. The input proteins in lanes 1 and 2 were from the hNSCs lysates and EVs extracted from hNSCs-culture supernatants, respectively. Antibodies targeting TSG101, Alix, and CD63 were used for immunoblotting EV membrane proteins, with calnexin used as the quality control marker. **B,** Transmission electron micrographs of EVs. Scale bar = 200 nm. **C,** Nanoparticle tracking analysis of EVs. **D,** Volcano plots of the differentially expressed mRNAs in the brain-derived EVs for the hNSCs versus control groups (left panel) and control versus healthy groups (middle panel). Grid plot (right panel) demonstrating the relationship of expression fold-changes between hNSCs versus control groups (hNSCs, vertical axis) and control versus healthy groups (Control, horizontal axis). **E,** Volcano plots of differentially expressed mRNAs in plasma-derived EVs for the hNSC versus control groups (left panel) and control versus healthy groups (middle panel). Grid plot (right panel) demonstrating the relationships of expression fold-changes between hNSCs versus control groups (hNSCs, vertical axis) and control versus healthy groups (Control, horizontal axis).

**Supplementary Figure 4. Differentially expressed miRNAs after hNSCs transplantation.**

**A,** Brain-derived, differentially expressed EV miRNAs of the hNSCs versus control groups at 4, 10, and 31 days after stroke. **B,** Plasma-derived, differentially expressed EVs miRNAs of the hNSCs versus control groups at 4, 10, and 31 days after stroke**. C,** The Upset plot illustrates the overlapping and distinct differentially expressed miRNAs in blood plasma samples, comparing mesenchymal stromal cell transplantation^1^ and hNSCs transplantation against their non-transplanted controls.

**Supplementary Figure 5. Joint analysis of brain and plasma-derived EV RNA molecules.**

**A,** Principal component analysis (PCA) plots of brain and plasma-derived EV RNA samples. The upper PCA feature space was constructed by miRNAs, whereas the bottom PCA feature space was constructed by mRNAs. **B,** Heatmap visualization of the top 50 most variably expressed genes across all brain or plasma-derived EV samples (left and right panels, respectively). The columns were split and categorized according to the time points and groups in which the samples were collected, and hierarchical clustering was performed within each sector. The genes of interest were listed in the row on the heatmaps. **C,** Density plots of brain-plasma miRNA correlations (upper panel) or mRNA correlations (bottom panel). **D,** Four brain-enriched miRNA expression levels in the brain and their correlated plasma levels. The red dots represent the hNSCs group, and the blue dots represent the stroke control group. The correlation coefficients were computed with Pearson correlation.

**Supplementary Figure 6. A,** Left panel: Pearson correlation plots of hub mRNAs in the pathway network. Right panel: Trend plots of the corresponding mRNAs. The line colors represent the origin of the sample types collected. **B,** Boxplots of hub mRNA expression in different groups. **C,** Left: GFAP^+^ astrocytes and Iba-1^+^ microglial cells in the infarct (*) and peri-infarct areas. The distance between the two dashed lines represents the width of the GFAP scar. Scale bar = 100 μm. Right: quantification of GFAP scar thickness and percentage of microglial areas between hNSCs and control groups (n= 5 per group). D , Veen diagram showing the identified target genes for miR-204-5p based on Tarbase, miRDB, and TargetScan. **E,** Flow cytometry analysis of EdU^+^ cell percentage in different groups *in vitro*. Data are presented as mean ± SD (n=3 biological replicates). Right: Quantification of the flow cytometry analysis result. Statistical significance was determined by one-way ANOVA followed by Student’s *t*-tests for pairwise comparisons, **P*<0.05, ***P* <0.01, ****P* <0.001.

**Supplementary Methods**

**Human neural stem cells (hNSCs) culture**

Primary hNSCs were obtained from the telencephalon of a human fetus at Beijing Yinfeng Dingcheng Biological Engineering Technology Co Ltd, Beijing, China.^2^ Ethical clearance for acquiring hNSCs was granted by the Ethics Committees of Peking Union Medical College Hospital (Approval No. 2021-01). The quality of hNSCs was confirmed by the National Medical Products Administration of China (SH202001141).

hNSCs were cultured in serum-free medium composed of DMEM/F12 (Gibco, Grand Island), supplemented with B-27 (Gibco), N-2 (Gibco), 20 ng/mL of recombinant human epidermal growth factor (EGF) (Invitrogen), 20 ng/mL of recombinant basic fibroblast growth factor (bFGF) (Invitrogen), and 8 μg/ml heparin (Sigma, St. Louis, MO). The majority of cells formed clusters of small, round cells, which developed into floating neurospheres and were dissociated every seven days. Subsequently, cells were characterized using flow cytometry with neural stem cell markers Nestin, Sox2, and CD133. To induce differentiation, mitogens were withdrawn from the culture medium. Neurospheres were dissociated into single cells and plated onto coated dishes with accutase medium (Sigma). Immunofluorescent staining revealed that hNSCs spontaneously differentiated into neural (Tuj1) and astro-glial (GFAP) lineages after 10 days of culture.

**Animal experiment**

Forty adult male Sprague-Dawley rats, aged 8 weeks and weighing 280-350 grams, were housed in a controlled environment maintained at 22 ± 1 °C with 50 ± 5% relative humidity and subjected to a 12-hour light/dark cycle commencing at 7:00. Rats were fed at 7:30, 12:00, and 15:00 daily. Prior to experimentation, they were anesthetized with 5% isoflurane, and their body temperature was rigorously maintained within the range of 37.0°C to 37.5°C. The rats then underwent transient middle cerebral artery occlusion (tMCAO). First, the midline skin in the neck was incised and retracted laterally to expose the subdermal structures. The right common carotid artery (CCA), external carotid artery (ECA), and internal carotid artery (ICA) were exposed. The ECA was then cut at its distal stem, and a silicon-coated nylon thread occluder was inserted into the bifurcation of the CCA and ICA to occlude the middle cerebral artery (MCA). The length of insertion of the nylon thread was roughly 20 mm to ensure that the occluder passed through the ICA rather than the pterygopalatine artery. After placing the occluder, it was fixed at the freed ECA endpoint with a surgical knot. After 90 minutes of occlusion, the rats were re-anesthetized, and the occluder was removed to allow reperfusion of the rats.

A longitudinal behavioral evaluation of stroke rats was conducted using the modified neurological severity score (mNSS), a quantitative scale encompassing motor, sensory, beam balance, and reflection tests.^3^ Motor tests assessed limb function, including tail-raising and walking ability. Sensory tests evaluated visual and tactile responses, as well as muscle stimulation. Beam balance tests measured balance maintenance, while reflection tests assessed neurological integrity. A longitudinal design with varying sample sizes (initially 15, then 12, and finally 9 animals per group) was employed, accounting for the 3 animals per group sacrificed for EV extraction at 4- and 10-days post-stroke. The adhesive removal test was performed to evaluate sensorimotor function in both groups (n=5 per group).^4^ Briefly, small adhesive patches (5 × 5 mm) were placed on the forepaws of each animal, and the time taken to detect (sensory function) and remove (motor function) the patches was recorded over three consecutive trials per session. The maximum allowed time per trial was 300 seconds. Testing was conducted before and after intervention to assess changes in sensorimotor performance. Data were analyzed as the mean latency to detect and remove the adhesive, with shorter latencies indicating better sensorimotor function. The Experimental Animal Welfare Ethics Committee of Peking Union Medical College Hospital approved all animal procedures (No. XHDW-2023-0320).

**hNSCs transplantation**

On the third day following post-stroke surgery, the rats' right CCAs were re-exposed. 2 × 10^5^ hNSCs in 0.2 mL glucose buffer or saline only were injected slowly into the rats' CCAs using a 31-gauge needle. For the imaging group, green fluorescent protein-labeled hNSCs (GFP-hNSCs; 2 × 10^5^ cells) were injected using the same techniques. To assess cell proliferation, bromodeoxyuridine (BrdU) (Sigma) was intraperitoneally injected into the animals at a concentration of 50 mg/kg, 12 days post-stroke induction. This injection was administered for three consecutive days before euthanasia.

**Mixed lymphocyte reaction assay**

To isolate lymphocytes, spleen tissue was gently ground and filtered through a 70 μm filter. The surface layer was mixed with phosphate-buffered saline (PBS) and centrifuged at 800g for 30 minutes. The middle layer containing lymphocytes was collected, washed with PBS, centrifuged again, and discarded into the supernatant. The collected cells were mixed with a CFSE Cell Division Tracker Kit (423801; BioLegend) and incubated in the dark for 20 minutes. CFSE is a green fluorescent dye that binds to proteins, and its fluorescence intensity decreases as the cells divide. The cells were mixed with RPMI 1640 medium (11875093; Thermo Scientific) containing 10% fetal bovine serum to terminate digestion. After centrifugation, the supernatant was discarded, and the cells were resuspended in RPMI 1640 complete medium and incubated for 10 minutes. The cells were then plated at a ratio of immune cells to neuronal cells of 3 × 10^5^cells / 1 × 10^5^cells and cultured at 37℃ with 5% CO_2_ for 5 days. After cultivation, the cells were collected and added with the following antibodies: APC anti-rat CD3 (201414; BioLegend), PE anti-rat CD4 (201507; BioLegend), APC anti-rat CD8a (200609; BioLegned), APC anti-rat CD25 (202114; BioLegend), APC anti-rat CD45RA (202313; BioLegend), PE anti-rat CD86 (200308; BioLegend), PE anti-rat CD161 (205604; BioLegend), and PE anti-rat IL-4 (511905; BioLegend). Samples were then incubated in the dark for 15 minutes at room temperature. For PE anti-rat IL-4 staining, 2 ml of Intracellular Staining Perm Wash Buffer (421002; BioLegned) was used before the addition of the antibody and incubated in the dark for 30 minutes at room temperature. The cells were then resuspended in PBS and centrifuged at 500g for 5 minutes; the supernatant was discarded, and the cells were resuspended in 100ul PBS for Flow cytometry analysis using a CytoFlex S instrument (Beckman Coulter company).

**Multiplex cytokine assay**

Supernatants were collected from lysed from rat serum samples using a RIPA lysis buffer. The samples were centrifugation at 13,000 rpm for 10 minutes at 4°C to clear cellular debris. A standard sample was prepared according to the manufacturer’s protocol. For each sample, 50 μL of supernatant was incubated with antibody-coupled fluorescent beads for 2 hours at room temperature with continuous shaking at 800 rpm. After incubation, the plates were washed twice with wash buffer to remove unbound proteins. 25 μL of biotinylated detection antibodies specific to each cytokine were added and incubated for 30 minutes at room temperature. Streptavidin–phycoerythrin conjugate was added to each well and incubated for an additional 30 minutes. Samples were analyzed using the Luminex-200 flow-based system (Luminex Corporation, Austin, TX, USA).

**Magnetic resonance imaging**

Magnetic resonance imaging (MRI) was acquired 2 and 31 days after the stroke onset. The experiment used a 9.4 T small animal MR scanner (Bruker BioSpec 94/20 MRI system). The rats were initially anesthetized with 5% isoflurane and maintained under anesthesia with 2-2.5% isoflurane throughout the MR scan. T2-weighted images were obtained using a rapid acquisition with relaxation enhancement (RARE) sequence, with the following imaging parameters: repetition time (TR) / echo time (TE) = 3137.94 / 33 ms, number of averages (NA) = 2, field of view (FOV) = 30 × 30 mm², image matrix size of 256 × 256, number of slices (NS) = 30, and slice thickness (ST) = 0.8 mm. Diffusion tensor imaging (DTI) images were acquired using a single shot spin echo-planar imaging sequence with the following parameters: TR / TE = 2000 / 22 ms, FOV = 25 × 25 mm², image matrix = 128 × 128, in-plane resolution = 0.195 × 0.195 × 0.4 mm³, diffusion encoding gradient directions = 30, gradient duration (δ) = 2.5 ms, gradient separations (Δ) = 8.5 ms, with b-value = 0 and 1000 s/mm².

All images were analyzed by researchers blinded to the experiment conditions. T2-weighted images were used to calculate the infarct volume of rats subjected to tMCAO. The T2-weighted MRI data were analyzed using the ITK-SNAP software (version 4.1.0) with a previously published protocol.^5^ The raw DTI was preprocessed using the following steps: 1) DICOM format images were converted to NIFTI format using MRIcroGL (dcm2nii tool, version 12.4). 2) The NIFTI format images were then imported to FSL to separate the brain from non-brain structures to create a brain mask. 3) The diffusion tensor was calculated at each voxel and a DTI index map for fractional anisotropy (FA), axial diffusivity (AD), and radial diffusivity (RD) was created using the FSL’s DTIFIT tool. A region of interest (ROI)-based analysis was used for analyzing the diffusion indices using DSI studio (<http://dsi-studio.labsolver.org>). The corpus callosum of the ipsilateral ischemic hemisphere was selected as the ROI. Contralateral homologous ROIs were similarly delineated in the contralateral hemisphere. Relative changes in FA (rFA), AD (rAD), and RD (rRD) values were computed by dividing the values of the ipsilateral ROI by those of the contralateral ROI, thus mitigating individual variability.

**Immunofluorescence staining and quantification**

Animals were transcardially perfused with PBS, followed by 4% paraformaldehyde. After removal, brains were fixed for 48 h and placed in 30% sucrose in PBS. Brains were then cryosectioned at 30 mm using a microtome. For fluorescent immunohistochemistry, sections were blocked in PBS 0.3% triton (HFH10; Invitrogen) with 5% goat serum (S1000; Vector Labs, Burlingame, CA, USA) and 1% bovine serum albumin (BSA; A2153; Sigma-Aldrich) for 1 h at room temperature. The primary antibody was incubated overnight (12–14 h) at 4°C in a 1:10 dilution of blocking solution (5% goat serum/1% BSA) with the following antibodies: anti-Nestin (ab221660; Abcam), anti-glial fibrillary acidic protein (GFAP; CST80788; Cell Signaling Technology), anti-CD31 (ab281583; Abcam), anti-doublecortin (Dcx; ab207175; Abcam), anti-β-III tubulin (Tuj-1; ab18207; Abcam), anti-ionized calcium-binding adaptor molecule 1 (Iba-1; CST17198; Cell Signaling Technology), anti-β-dystroglycan (βDG; 66735-1-IG; Proteintech), anti- 5′-bromo-2′-deoxyuridine (BrdU; 59-14-3; Sigma-Aldrich), anti-green fluorescent protein (GFP; AF10669; Afantibody).

The survival and differentiation of exogenous stem cells in rat brains were analyzed in the group treated with GFP-hNSCs using a mouse monoclonal anti-GFP antibody. For the analysis of endogenous cell proliferation and differentiation of newborn cells, BrdU (50 mg/kg body weight) was injected into the rats once daily via the intraperitoneal route from day 12 to 14. Primary antibodies were detected using appropriate Cy-3-labeled, Alexa Fluor 594-labeled, or Alexa Fluor 488-labeled secondary antibodies (Invitrogen) at 4°C for 6 hours. The sections were then stained with DAPI (Invitrogen) at a concentration of 1:2000. The stained sections were visualized on a laser scanning confocal microscope (NIKON ECLIPSE C1, Japan).

The ischemic penumbra in the cortex was selected as the region of interest (ROI). The scar thickness was measured on the ischemic boundary zone within the ipsilateral hemisphere on four sections stained for GFAP. The quantification of vascular density was carried out on the ischemic penumbra within the ipsilateral hemisphere stained for endothelial marker CD31. The neuroblast migration was determined by Dcx staining on the ipsilateral hemisphere, representing the length of migration of Dcx-positive neuroblasts along the corpus callosum.

Statistical analysis of non-omics datasets involved using students' t-tests (two-tailed) to compare two groups. For multiple group comparisons analysis of variance (ANOVA), followed by Holm-Sidak post-hoc multiple comparison tests, were used to evaluate statistically significant differences.

**Extracellular vesicle isolation**

The plasma EVs and hNSCs-secreted EVs were isolated using size exclusion chromatography (SEC) techniques, with some minor modifications as described previously by Boing et al.^6^ In brief, 1 mL of blood plasma or 200 mL hNSC-cultured supernatant filtered through a 0.8 μm filter was diluted 1.5-fold with PBS. The diluted plasma was then passed through Exosupur^®^ columns (Echobiotech, China) for further purification. The samples were eluted with an additional 0.1 M PBS, and a total of 2 mL eluate fractions were collected according to the manufacturer’s instructions. The fractions were then concentrated to 200 μL by centrifugation through Amicon^®^ Ultra spin filters with a 100 kDa molecular weight cut-off (Merck, Germany). The isolation of EVs from brain tissue was performed using a modified protocol originally described by Vella et al.^7^ The tissue was first dissociated using the Miltenyi Human Tumor Dissociation Kit (Miltenyi Biotec) with enzymes H, R, and A. Before use, the enzymes were resuspended according to the manufacturer's instructions. The dissociation mix, containing RPMI media, enzymes H, R, and A, was prepared fresh for each use. A small piece of tissue (~200 mg) was weighed, briefly sliced on dry ice, and then incubated in the dissociation mixture for 10-15 minutes at 37°C. The tissue was filtered through a 70 μm filter twice to remove residual tissue fragments. The suspension was then centrifuged at 300 × g for 10 minutes at 4°C, and the supernatant was transferred to a fresh tube. It was then centrifuged at 2000 × g for 10 minutes at 4°C. The supernatant was spun at 10,000 × g for 20 minutes at 4°C, and filtered through a 0.22 μm filter to remove any remaining cell debris. The suspension was then processed by ultracentrifugation (UC) at 150,000 × g for 2 hours at 4°C. The resulting pellet was resuspended in 1 mL PBS and further purified using Exosupur^®^ columns (Echobiotech, China). The fractions were concentrated to 200 μL using Amicon^®^ Ultra spin filters with a 100 kDa molecular weight cut-off (Merck, Germany).

**Nanoparticle tracking analysis**

The concentration of vesicles in suspension was between 1x10^7^/ml and 1x10^9^/ml. These suspensions were then analyzed using the ZetaView PMX 110 instrument (Particle Metrix, Meerbusch, Germany) equipped with a 405 nm laser. A 60-second video was captured at a frame rate of 30 frames per second, and the movement of particles was analyzed using the nanoparticle tracking analysis software (ZetaView 8.02.28). This allowed for the determination of particle size and quantity in the suspensions.

**Transmission electron microscopy**

A 10 µl aliquot of the EV solution was placed on a copper mesh and incubated at room temperature for 1 minute. After washing with sterile distilled water, the copper mesh was contrasted with uranyl acetate solution for an additional minute. The sample was then dried under an incandescent light for 2 minutes. The copper mesh was then observed and photographed using a transmission electron microscope (H-7650, Hitachi Ltd., Tokyo, Japan).

**Western blot analysis**

The EV supernatant was denatured in 5× sodium dodecyl sulfonate (SDS) buffer and then subjected to western blot analysis. This analysis involved electrophoresis on a 10% SDS-polyacrylamide gel, with 50 µg protein loaded per lane. The separated proteins were then transferred to a polyvinylidene difluoride membrane. The membrane was blocked with 5% non-fat milk in Tris-buffered saline containing 0.1% Tween-20. For rat EVs, membranes were probed with rabbit monoclonal TSG101 (ab125011, 1:1000, Abcam), rabbit monoclonal CD9 (ab92726, 1:1000, Abcam), rabbit monoclonal HSP70 (ab181606, 1:1000, Abcam), and rabbit polyclonal Calnexin (10427-2, 1:500, Proteintech). For human neural stem cell EVs, rabbit monoclonal TSG101 (ab125011, 1:1000, Abcam), rabbit monoclonal Alix (ab186429, 1:1000, Abcam), mouse monoclonal CD63 (sc-5275, 1:200, Santa), and rabbit polyclonal Calnexin (10427-2, 1:500, Proteintech) were used for membrane probing. For in vitro experiment, the Anti-ephrin-B3 (sc-271328, 1:1,000, Santa Cruz Biotechnology) and β-actin(20536-1-AP,1:1000, Proteintech). The membrane was then washed and incubated with the appropriate secondary antibody at room temperature for 2 hours. The bands were visualized using the Tanon4600 chemiluminescence detection system and the BioSpectrum Imaging System (UVP, CA, USA).

**RNA isolation from EVs and library preparation**

The total RNA was extracted and purified from EVs using the miRNeasy Advanced Kit (Qiagen, cat. No. 217204) according to the kit instructions. The RNA concentration and purity were then evaluated using the RNA Nano 6000 Assay Kit on the Agilent Bioanalyzer 2100 System (Agilent Technologies, CA, USA). For long RNA library preparation, 250pg-10ng RNA per sample was used as input material for libraries created with the SMARTer Stranded Total RNA-Seq Kit (Takara Bio USA, Inc.) following the manufacturer's recommendations. Index codes were added to attribute sequences to each sample. A range of 1ng-500ng RNA per sample was used as input material for small RNA libraries. Sequencing libraries were generated using the QIAseq miRNA Library Kit (Qiagen, Frederick, MD), with index codes and unique molecule identifier sequences added to each small RNA fragment. The quality of the libraries was assessed using the Fragment Analyzer Qseq100 and qPCR. The indexed samples were clustered on the acBot Cluster Generation System using the TruSeq PE Cluster Kitv3-cBot-HS (Illumina, San Diego, CA, USA). After cluster generation, the libraries were sequenced on the Illumina Novaseq 6000 platform, generating paired-end reads.

The raw data in fastq format were first processed using in-house Perl scripts. This step involved removing adapter sequences, poly-N-containing reads (reads containing at least 10% of unknown bases), and low-quality reads (reads with a proportion of bases below Q30 exceeding 20%) from the raw data. Reads with a length of less than 18 nucleotides (or greater than 30 nucleotides) after adapter removal were excluded. All subsequent analyses were based on this high-quality, clean data. Paired-end clean reads were then aligned to the reference genome Rnor_6.0 or GRCh38.p10 using Bowtie2.^8^ The mapped reads were used for gene expression level quantification and differential expression analysis. Stringtie was employed to calculate the fragments per kilo-base of exon per million fragments mapped (FPKMs) of coding genes in each sample. Gene FPKMs were computed by summing the FPKMs of transcripts in each gene group. FPKM is a measure based on fragment length and read count mapped to that fragment.

Using Bowtie tools, small RNA clean reads were aligned to Silva, GtRNAdb, Rfam, and Repbase databases. Sequences were filtered to remove ribosomal RNA (rRNA), transfer RNA (tRNA), small nuclear RNA (snRNA), small nucleolar RNA (snoRNA), and other non-coding RNAs and repeats. The remaining reads were then used to detect known miRNA from miRbase. Read counts for each miRNA were obtained from the mapping results, and tags per million (TPM) values were calculated. A threshold of TPM >5 was applied to filter out lowly expressed miRNAs.

**Transcriptome analyses**

The R package limma (3.52.4) was used to identify mRNAs and miRNAs that were differentially expressed, with an absolute fold change > 1.5 and a P value < 0.05, adjusted by the Benjamini-Hochberg method.^9^ To evaluate the homology between *Rattus norvegicus* and *Homo sapiens* miRNAs, we counted the number of mismatches or nucleotide shifts in the miRNA sequences. Lower numbers of mismatches or nucleotide shifts indicate higher degrees of homology. We employed the R package multiMiR (2.3.0) to identify miRNA target genes in the *Rattus norvegicus* dataset.^10^ Furthermore, we used clusterProfiler (4.8.2) to conduct gene enrichment analysis to retrieve significantly enriched pathways from Gene Ontology (GO), Kyoto Encyclopedia of Genes and Genomes (KEGG), and Reactome, which were based on *Rattus norvegicus* annotations.^11^ Pathways were considered significantly enriched if their P values were ≤0.05. GO term enrichment encompassed biological processes, molecular functions, and cellular components across all GO levels.

The R package GSVA was used with default settings to calculate Z-scores.^12^ The gene set for this analysis was derived from mRNAs detected in EVs of hNSCs, and the expression data was based on mRNA from plasma-derived EVs aligned to the GRCh38.p10 human genome. To identify co-occurring expression alterations in the mRNA data, we employed the Mfuzz (2.56.0) package using the fuzzy c-means algorithm with a minimum standard deviation of > 0.5.^13^ For tissue-specific gene enrichment analysis, we utilized TissueEnrich (1.16.0) and GSE53960 for Rat tissue data, assigning tissue origins to EVs of different condition groups.^14, 15^ For source tracking of brain-derived EVs, we utilized scMappR (1.16.0) for the deconvolution process and GSE250245 for Rat brain single-cell transcriptomic data for constructing brain cell type signature matrix.^16, 17^ For visualizing our data, we created heatmap plots with ComplexHeatmap (2.12.1), volcano plots, boxplots, and bubble plots with ggplot2 (3.4.3),^18^ upset plots using UpSetR,^19^ and miRNA-gene network plots with Cytoscape software (v3.9.1, https://cytoscape.org/).

**Cell culture, OGD/R induction, and transfection**

HT22 hippocampal neuronal cells were maintained in high-glucose Dulbecco’s Modified Eagle Medium (DMEM; 4.5 g/L glucose) supplemented with 10% fetal bovine serum (FBS) and 1% penicillin-streptomycin at 37°C under 5% CO₂. For oxygen-glucose deprivation/reoxygenation (OGD/R) modeling, cells were washed twice with PBS, and the medium was replaced with glucose-free DMEM. Cultures were then transferred to an anaerobic chamber infused with a gas mixture of 2% O₂, 5% CO₂, and 93% N₂ for 6 hours. After OGD, the glucose-free medium was replaced with standard high-glucose DMEM, and cells were returned to a normoxic incubator for 24 hours of recovery. For in vitro co-culture, human neural stem cells (hNSCs) were seeded into transwell inserts (0.8 μm pore size; Corning) at a density of 5 × 10⁴ cells/insert and positioned above the HT22 monolayer for an additional 24 hours. In cell transfection experiments, HT22 cells were transfected with either a miR-204-5p inhibitor or negative control (50 nM; Tsingke, China) using Lipofectamine 3000 (Thermo Fisher, USA) immediately after reoxygenation. Cells were harvested 48 hours post-transfection for further analysis.

**Cell apoptosis and proliferation assays**

HT22 cells from each experimental group were harvested using 0.25% EDTA-free trypsin (Gibco, USA), centrifuged, and washed twice with pre-cooled PBS. For apoptosis assessment, cells were resuspended in 100 μL binding buffer (1 × 10⁶ cells/mL), stained with 5 μL Annexin V-FITC and 5 μL PI (Invitrogen, USA), and incubated for 15 min in the dark. Apoptotic populations—viable (Annexin V⁻/PI⁻), early apoptotic (Annexin V⁺/PI⁻), late apoptotic (Annexin V⁺/PI⁺), and necrotic (Annexin V⁻/PI⁺)—were quantified within 1 h using an Attune NxT flow cytometer (Thermo Fisher, USA) and analyzed with FlowJo (v10.8.1). For proliferation analysis, cells from each experimental group were incubated with EdU for 1 hour at 37°C to evaluate proliferation rates, using the EdU Cell Proliferation Kit with Alexa Fluor 488 (Beyotime, China). Fluorescence images were captured using a Nikon Eclipse fluorescence microscope (Nikon, Japan). The number of EdU-positive cells (green) relative to the total nuclei (blue) was quantified from three randomly selected fields using ImageJ software (Version 1.53). For flow cytometry, EdU-labeled cells were analyzed using a Attune NxT flow cytometer (Thermo Fisher, USA), and data were analyzed using FlowJo software (version 10.8.1).

**Dual luciferase assay**

The predicted binding site of miR-204-5p in the EFNB3 3′-UTR was cloned and inserted into a pmirGLO vector to create the EFNB3 WT construct. The corresponding mutant, EFNB3 MT, was generated by site-directed mutagenesis. HEK 293 cells were transfected with pmirGLO vectors and miR-204-5p mimic/negative control using Lipofectamine 3000 (Thermo Fisher). 48 hours post-transfection, firefly and Renilla luciferase activities were quantified using the using the Dual-Luciferase Reporter Gene Assay Kit (Promega). Firefly luciferase activity was normalized to Renilla luciferase activity for each well. Data are expressed as relative luciferase activity (Firefly/Renilla).

**RT-qPCR**

Total RNA was extracted using TRIzol reagent (TIANGEN) according to the manufacturer’s instructions. The RNA was then reverse transcribed into cDNA using an all-in-one cDNA synthesis supermix (Tsingke) and a miRNA first-strand cDNA synthesis reagent kit by tailing A (Tsingke). mRNA and miRNA expression levels were quantified using SYBR Green Master Mix (Yeason). Relative expression levels were normalized to U6, which served as the internal reference. Primer sequences are provided in Table S9.

**Supplementary Tables**

Tables S1-S8 are uploaded separately in Excel file format.

Table S9. Primer sequences used for qRT-PCR.

| Gene |  |  |
| --- | --- | --- |
| rno-miR-653-5p | forward | GGTGCTGAAACAATCTCTACTGAAA |
| rno-miR-1298 | forward | TTCGGCTGTCCAGATGTACC |
| rno-miR-7a-5p | forward | GCTGGAAGACTAGTGATTTTGTTGT |
| rno-miR-7b | forward | CGAGGCAGTGTAATTAGCTGATTGT |
| rno-miR-34b-5p | forward | TATACCAGGATGTCAGCATAGTT |
| rno-miR-1949 | forward | TTCCCTTTGTCATCCTATGCCT |
| rno-miR-204-5p | forward | AGGCAGTGTAGTTAGCTGATTGC |
| rno-miR-34c-5p | forward | TGTCTTGCAGGCCGTCATG |
| rno-miR-3547 | forward | GCGTTTATTGAGCACCTCCTATC |
| rno-miR-431 | forward | GGTGCTGAAACAATCTCTACTGAAA |
| rno-miR-325-3p | forward | TTCGGCTGTCCAGATGTACC |
| EFNB3 | forward | AGTTCCGATCCCACCACGATTACT |
|  | reverse | AGAAGCACCTTCATGCCTCTGGTT |
| U6 | forward | CTCGCTTCGGCAGCACA |
|  | reverse | AACGCTTCACGAATTTGCGT |

**REFERENCES**

1. Huang L, Hua L, Zhang X. The Exosomal MicroRNA Profile Is Responsible for the Mesenchymal Stromal Cell Transplantation-Induced Improvement of Functional Recovery after Stroke in Rats. *Neuroimmunomodulation*. 2021;29(2):151-160.

2. Luan Z, Liu W, Qu S, et al. Effects of neural progenitor cell transplantation in children with severe cerebral palsy. *Cell Transplant*. 2012;21 Suppl 1:S91-8.

3. Li Y, Chen J, Wang L, Lu M, Chopp M. Treatment of stroke in rat with intracarotid administration of marrow stromal cells. *Neurology*. 2001;56(12):1666-72.

4. Bouet V, Boulouard M, Toutain J, et al. The adhesive removal test: a sensitive method to assess sensorimotor deficits in mice. *Nat Protoc*. 2009;4(10):1560-4.

5. Yushkevich PA, Piven J, Hazlett HC, et al. User-guided 3D active contour segmentation of anatomical structures: significantly improved efficiency and reliability. *Neuroimage*. 2006;31(3):1116-28.

6. Böing AN, van der Pol E, Grootemaat AE, et al. Single-step isolation of extracellular vesicles by size-exclusion chromatography. *J Extracell Vesicles*. 2014;3

7. Vella LJ, Scicluna BJ, Cheng L, et al. A rigorous method to enrich for exosomes from brain tissue. *J Extracell Vesicles*. 2017;6(1):1348885.

8. Langmead B, Salzberg SL. Fast gapped-read alignment with Bowtie 2. *Nat Methods*. 2012;9(4):357-9.

9. Ritchie ME, Phipson B, Wu D, et al. limma powers differential expression analyses for RNA-sequencing and microarray studies. *Nucleic Acids Res*. 2015;43(7):e47.

10. Ru Y, Kechris KJ, Tabakoff B, et al. The multiMiR R package and database: integration of microRNA-target interactions along with their disease and drug associations. *Nucleic Acids Res*. 2014;42(17):e133.

11. Wu T, Hu E, Xu S, et al. clusterProfiler 4.0: A universal enrichment tool for interpreting omics data. *Innovation (Camb)*. 2021;2(3):100141.

12. Hänzelmann S, Castelo R, Guinney J. GSVA: gene set variation analysis for microarray and RNA-seq data. *BMC Bioinformatics*. 2013;14:7.

13. Kumar L, M EF. Mfuzz: a software package for soft clustering of microarray data. *Bioinformation*. 2007;2(1):5-7.

14. Jain A, Tuteja G. TissueEnrich: Tissue-specific gene enrichment analysis. *Bioinformatics*. 2019;35(11):1966-1967.

15. Yu Y, Fuscoe JC, Zhao C, et al. A rat RNA-Seq transcriptomic BodyMap across 11 organs and 4 developmental stages. *Nat Commun*. 2014;5:3230.

16. Ma S, Sun S, Geng L, et al. Caloric Restriction Reprograms the Single-Cell Transcriptional Landscape of Rattus Norvegicus Aging. *Cell*. 2020;180(5):984-1001.e22.

17. Sokolowski DJ, Faykoo-Martinez M, Erdman L, et al. Single-cell mapper (scMappR): using scRNA-seq to infer the cell-type specificities of differentially expressed genes. *NAR Genom Bioinform*. 2021;3(1):lqab011.

18. Ginestet C. ggplot2: Elegant Graphics for Data Analysis. *Journal of the Royal Statistical Society Series A: Statistics in Society*. 2011;174(1):245-246.

19. Conway JR, Lex A, Gehlenborg N. UpSetR: an R package for the visualization of intersecting sets and their properties. *Bioinformatics*. 2017;33(18):2938-2940.
